# First use of anatomical networks to study modularity and integration of heads, forelimbs and hindlimbs in abnormal anencephalic and cyclopic *vs* normal human development

**DOI:** 10.1038/s41598-019-44314-z

**Published:** 2019-05-24

**Authors:** Rui Diogo, Janine M. Ziermann, Christopher Smith, Malak Alghamdi, Jose S. M. Fuentes, Andre Duerinckx

**Affiliations:** 1 Department of Anatomy, Howard University College of Medicine, Washington, D.C. USA; 2 Graduate Center, City University of New York, New York, USA; 3 Kumangui research group, Univ. Francisco Jose de Caldas, Bogotá, Colombia; 4 Department of Radiology, Howard University College of Medicine, Washington, D.C. USA; 5 College of Medicine, King Saud bin Abdulaziz University for Health Sciences, Jeddah, Saudi Arabia; 6 King Abdullah International Medical Research Center, Jeddah, Saudi Arabia

**Keywords:** Biological anthropology, Evolutionary developmental biology

## Abstract

The ill-named “logic of monsters” hypothesis of Pere Alberch - one of the founders of modern evo-devo - emphasized the importance of “internal rules” due to strong developmental constraints, linked teratologies to developmental processes and patterns, and contradicted hypotheses arguing that birth defects are related to a chaotic and random disarray of developmental mechanisms. We test these hypotheses using, for the first time, anatomical network analysis (AnNA) to study and compare the musculoskeletal modularity and integration of both the heads and the fore- and hindlimbs of abnormal cyclopic trisomy 18 and anencephalic human fetuses, and of normal fetal, newborn, and adult humans. Our previous works have shown that superficial gross anatomical analyses of these specimens strongly support the “logic of monsters” hypothesis, in the sense that there is an ‘order’ or ‘logic’ within the gross anatomical patterns observed in both the normal and abnormal individuals. Interestingly, the results of the AnNA done in the present work reveal a somewhat different pattern: at least concerning the musculoskeletal modules obtained in our AnNA, we observe a hybrid between the “logic of monsters” and the “lack of homeostasis” hypotheses. For instance, as predicted by the latter hypothesis, we found a high level of left-right asymmetry in the forelimbs and/or hindlimbs of the abnormal cyclopic trisomy 18 and anencephalic human fetuses. That is, a network analysis of the organization of/connection between the musculoskeletal structures of these fetuses reveals a more “chaotic” pattern than that detected by superficial gross anatomical comparisons. We discuss the broader developmental, evolutionary, and medical implications of these results.

## Introduction

In a classic work by Pere Alberch^1^, teratologies are discussed as forms that lack adaptive function, while preserving structural order, which was ill-named as “the logic of monsters”. This view opened a new window into the analysis of how multicellular organisms develop following “internal rules.” In fact, Alberch, as one of the founders of modern evo-devo, championed the importance of internalism as a valid framework in a developmentally-driven evolutionary process, where the action of external natural selection is a complementary - but still important - evolutionary force^2^. In this context, comparing teratological and normal forms can highlight developmental constraints that impact the evolvability of forms. Indeed, as pointed out by Alberch, teratologies are amongst the best tools to analyze developmental systems and to understand morphological diversity and variations. Due to strong developmental constraints and thus a limited set of possible phenotypic outcomes, a teratological form has to follow the same rules that make up a normal individual, rules that pertain to the developmental mechanisms available.

This idea has been advocated in the classic works by Étienne Geoffroy Saint-Hilaire and his son Isidore, who published seminal classifications of teratologies^3^. The introductory remark by Wright and Wagner^4^, who systematically analyzed naturally occurring teratologies in the guinea pig summarizes the history and importance of the studies of teratologies: “abnormalities have long attracted the interest of morphologists as natural experiments whose study should throw some light on the developmental process”. In the middle of the seventeenth century, Harvey interpreted harelip as due to arrest of development. In the eighteenth century, von Haller interpreted *ectopia cordis*, and Wolff, *exomphalos* with developmental bases, and this view was extended to other types of conditions by later authors^4^. Since then, anatomical and genetic accounts of teratologies have been studied at length. Moreover, in modern molecular biology, this kind of analysis is carried out systematically when performing mutagenesis, in which experimentally induced gene misexpression in model organisms allows the experimental exploration of the limits of developmental programs. The morphological variation thus obtained can be analyzed in various ways, from the obvious comparative analysis of the disappearance of morphological structures to more detailed geometric morphometrics analysis of shape and size differences. Despite the gross anatomical differences found in teratological vs. normal forms, as in cyclopia and/or trisomy 18 (T18), some measure of similarity can thus be predicted, as evidence of the same underlying developmental mechanisms at work.

However, such predictions, and Alberch’s “logic of monsters” in general, were never tested using explicit quantitative tools comparing, for instance, the overall configuration, number, connections, and modularity/integration of musculoskeletal structures in the normal vs. abnormal condition. Thus, the major goal of the present work is to use a new tool, Anatomical Network Analysis (AnNA), to test the hypothesis that the structural properties of the network patterns found in the normal adult, newborn, and fetal structures remain similar in human T18 cyclopic and anencephalic fetuses, or are instead more ‘chaotic’^5^.

AnNA is a formal framework to study morphological organization free of *a priori* assumptions about developmental, functional, and phylogenetic relationships among structures. As such, AnNA was used to provide new insights on the musculoskeletal organization of the adult human head, on the study of birth defects such as premature fusion of skull bones or craniosynostosis or forelimb (FL) defects, on the evolution of the primate heads and FL, and of the chimpanzee-human hindlimbs (HLs) and FLs^6–15^. AnNA evaluates the connectivity patterns that define the morphological organization of anatomies using tools and statistics borrowed from network theory^12,13^. For any anatomical system, AnNA formalizes bones, muscles, and their physical contacts as the nodes and links of a network model, using computational methods to assess the morphological organization of the system and to identify patterns of integration and modularity among muscles and bones.

This is therefore the first application of anatomical networks to study the musculoskeletal modularity and integration of the head and limbs of both T18 cyclopic and anencephalic human fetuses, and compare them with those seen in the normal newborn, fetal, and adult human heads and limbs. Using AnNA allows us a glimpse into the morphological integration patterns resulting from a perturbed genetic condition causing severe phenotypic malformations. Cyclopia (one eye or partially fused eyes) is linked to the failure of the brain to separate into distinct hemispheres during development and often co-presents with *alobar holoprosencephaly*: authors often use the phrase “the face predicts the brain” to describe how the developing brain has a strong influence on the developing surrounding structures^16,17^. Anencephaly results from failure of the cranial portion of the neural tube to fuse, resulting in partial or complete absence of cranial vault (frontal, parietal, occipital, temporal, sphenoid, and ethmoid bones) with absence of overlying tissues and malformation and damage of fundamental brain structures (recently reviewed by Alghamdi *et al*.^18^). The present study will therefore provide new data to discuss these and other broader developmental and evolutionary hypotheses and to better understand the links between normal and abnormal development, morphological diversity, variations and defects, and modularity and integration.

## Results

**Figure 1:**
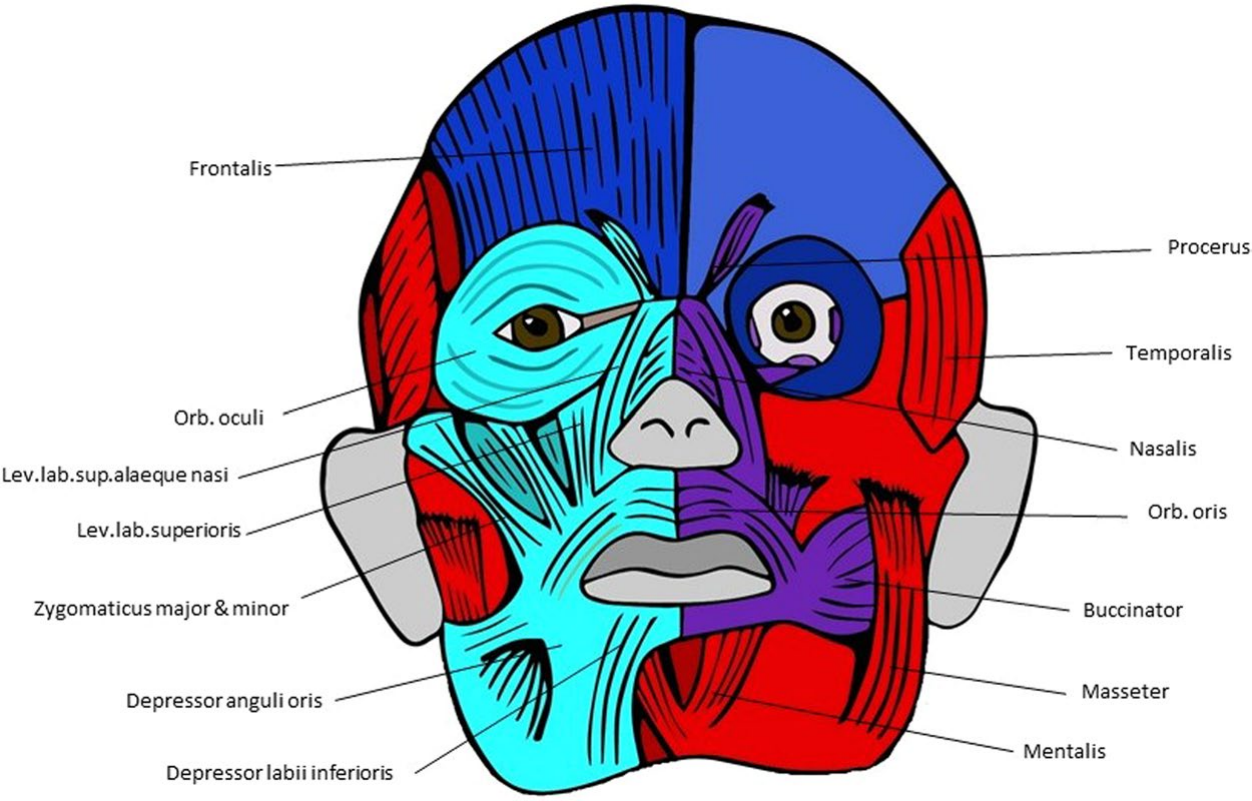
Musculoskeletal modules of the normal adult head identified using AnNA. In *blue*, the module 1 of Table 3 (“occipital”); in *red*, the module 3 of Table 3 (“eye/mastication”); in *turquoise*, the module 4 of Table 3 (“ocular/orofacial left”); in *purple*, the module 5 of Table 3 (“ocular/orofacial right”) (other modules of Table 3 not show here).

**Figure 2:**
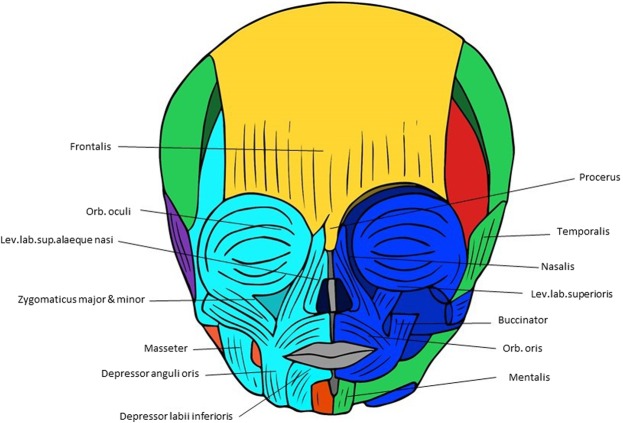
Musculoskeletal modules of the normal newborn head identified using AnNA. In *purple*, the module 1 of Table 4 (“occipital”); in *green*, the module 2 of Table 4 (“right facial-mastication-tongue”); in *orange*, the module 3 of Table 4 (“tongue”); in *turquoise*, the module 4 of Table 4 (“left eye-facial-oral-mastication”); in *blue*, the module 5 of Table 4 (“right eye-facial-oral”); in *red*, the module 6 of Table 4 (“eye-mastication”); in *yellow*, the module 7 of Table 4 (“frontalis + procerus”) (other modules of Table 4 not show here).

**Figure 3:**
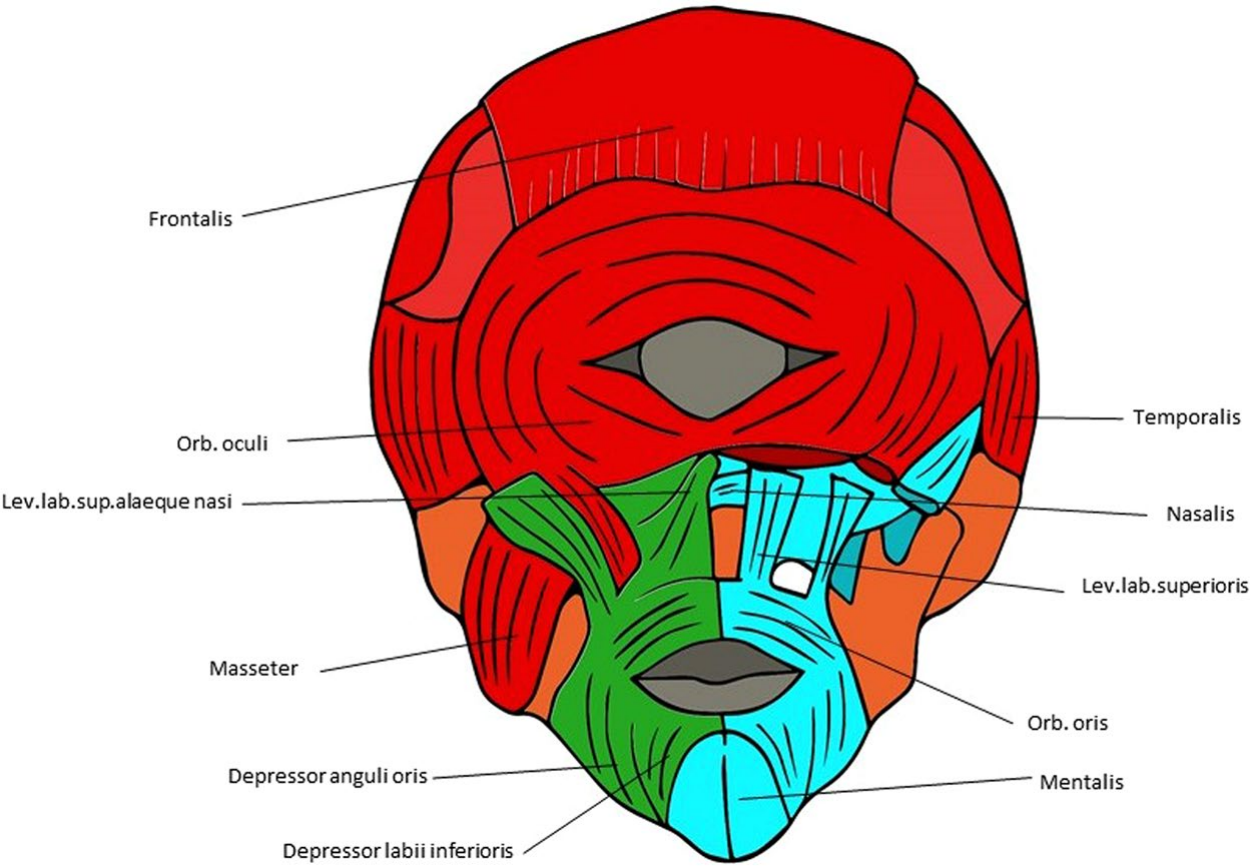
Musculoskeletal modules of the T18 cyclopic head identified using AnNA. In *orange*, the module 1 of Table 6 (“eye-mastication”); in *red*, the module 2 of Table 6 (“skull-mastication-facial”); in *green* and *light blue*, the modules 3 and 4 of Table 6 (“left and right facial”, respectively); in *dark blue*, the module 5 of Table 6 (“frontalis + occipitalis”) (other modules of Table 6 not show here).

**Figure 4:**
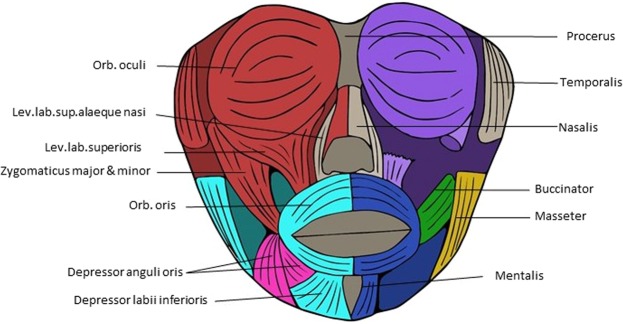
Musculoskeletal modules of the anencephalic fetus 1 head identified using AnNA. In *red*, the module 1 of Table 7 (“left upper-mid face”); in *turquoise*, the module 2 of Table 7 (“left skull-mandible-lower face”); in *blue*, the module 3 of Table 7 (“right mandible-lower face”); in *purple*, the module 4 of Table 7 (“right upper-mid face”); in *orange*, the module 5 of Table 7 (“skull-digastric-neck muscles”); in *pink*, the module 6 of Table 7 (“left risorius + left depressor anguli oris + platysma”); in *green*, the module 9 of Table 7 (“buccinator right”); in *yellow*, the module 10 of Table 7 (“masseter right”) (other modules of Table 7 not show here).

**Figure 5:**
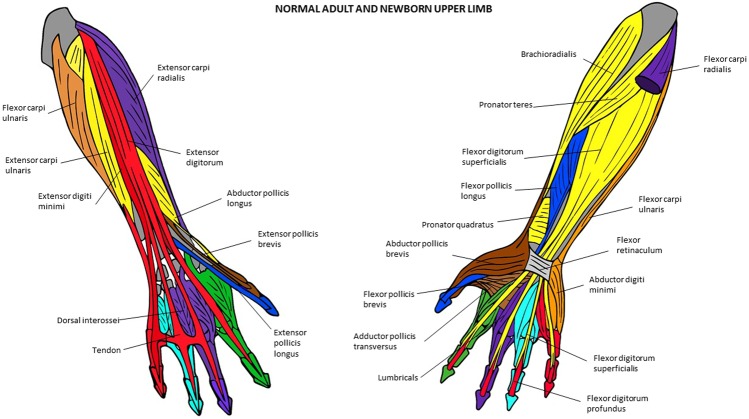
Musculoskeletal modules of the normal adult and newborn forelimb identified using AnNA. Dorsal view on the left side, ventral view on the right. In *yellow*, the module 2 of Tables 9 and 10 (“arm/forearm”); in *purple*, the module 3 of Table 9 and module 4 of Table 10 (“3rd digit”); in *orange*, the module 4 of Table 9 and module 3 of Table 10 (“carpals/digit 5”); in *red*, the module 5 of Tables 9 and 10 (“5th digit”); in *brown*, the module 6 of Tables 9 and 10 (“carpus/1st digit”); in *turquoise*, the module 7 of Tables 9 and 10 (“4th digit”); in *green*, the module 8 of Tables 9 and 10 (“2nd digit”); in *blue*, the module 9 of Tables 9 and 10 (“distal phalanx 1”) other modules of Tables 9 and 10 not show here).

**Figure 6:**
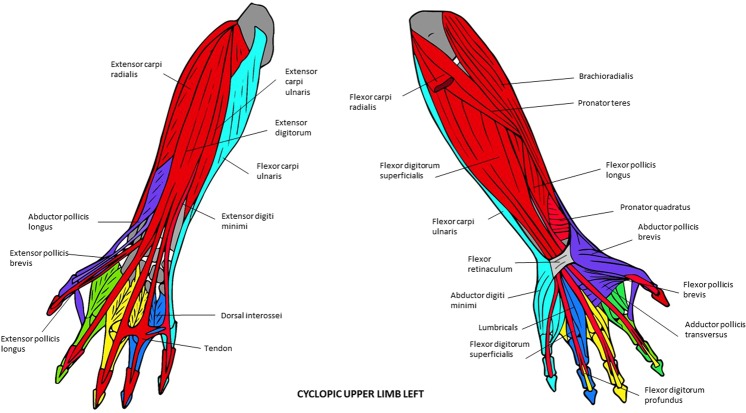
Musculoskeletal modules of the left forelimb of the T-18 cyclopic fetus identified using AnNA. Dorsal view on the left side, ventral view on the right. In *turquoise*, the module 1 of Table 13 (“carpus/5th digit”); in *red*, the module 3 of Table 13 (“arm/forearm/1st digit”); in *purple*, the module 5 of Table 13 (“carpus/1st digit”); in *yellow*, the module 6 of Table 13 (“3rd digit”); in *blue*, the module 7 of Table 13 (“4th digit”); in *green*, the module 8 of Table 13 (“2nd digit”) (other modules of Table 13 not show here).

**Figure 7:**
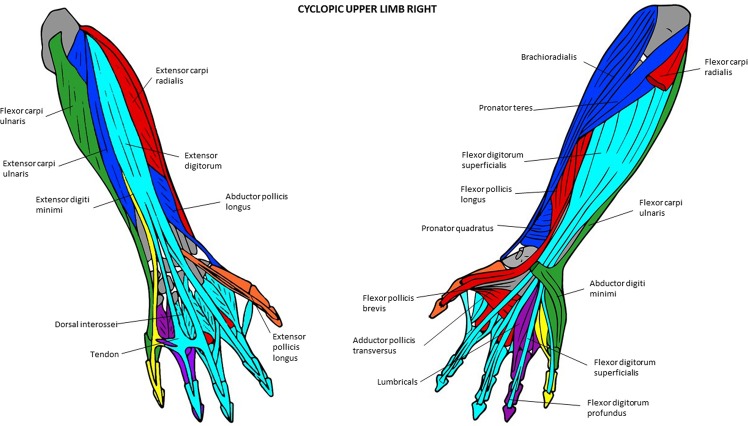
Musculoskeletal modules of the right forelimb of the T-18 cyclopic fetus identified using AnNA. Dorsal view on the left side, ventral view on the right. In *turquoise*, the module 2 of Table 14 (“2nd and 3rd digits”); in *blue*, the module 3 of Table 14 (“shoulder girdle/arm/forearm”); in *green*, the module 4 of Table 14 (“carpus/5th digit”); in *orange*, the module 5 of Table 14 (“1st digit”); in *red*, the module 6 of Table 14 (“carpus/metacarpus “); in *purple*, the module 8 of Table 14 (“4th digit”); in *yellow*, the module 9 of Table 14 (“5th digit”) (other modules of Table 14 not show here).

**Figure 8:**
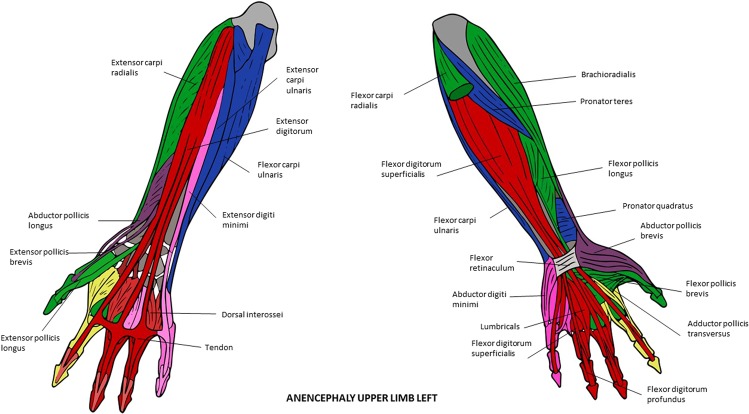
Musculoskeletal modules of the left forelimb of the anencephaly fetus 1 identified using AnNA. Dorsal view on the left side, ventral view on the right. In *green*, the module 1 of Table 15 (“carpus/1st digit”); in *blue*, the module 2 of Table 15 (“shoulder girdle/arm/forearm”); in *pink*, the module 3 of Table 15 (“carpus/5th digit”); in *red*, the module 4 of Table 15 (“3rd & 4th digits”); in *purple*, the module 6 of Table 15 (“pollicis”); in *yellow*, the module 7 of Table 15 (“2nd digit”) (other modules of Table 15 not show here).

**Figure 9:**
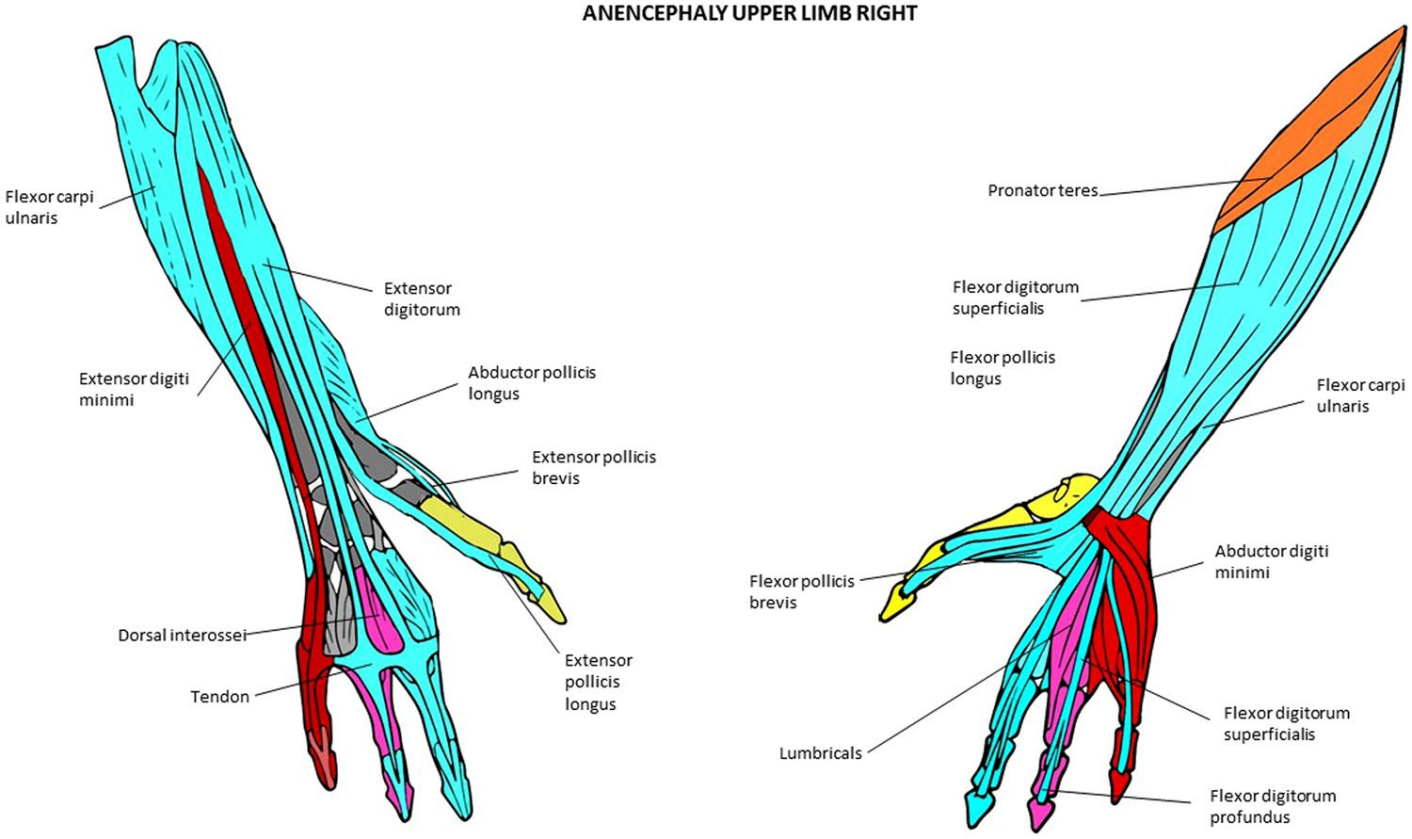
Musculoskeletal modules of the right forelimb of the anencephaly fetus 1 identified using AnNA. Dorsal view on the left side, ventral view on the right; note that the metacarpal 2 and all phalanges of digit 2 are absent or very reduced in size (see text and SI). In *turquoise*, the module 1 of Table 16 (“carpus/3rd digit”); in *orange*, the module 2 of Table 16 (“shoulder girdle/arm”); in *red*, the module 3 of Table 16 (“carpus/5th digit”); in *yellow*, the module 4 of Table 16 (“1st digit”); in *pink*, the module 6 of Table 16 (“4th digit”).

**Figure 10:**
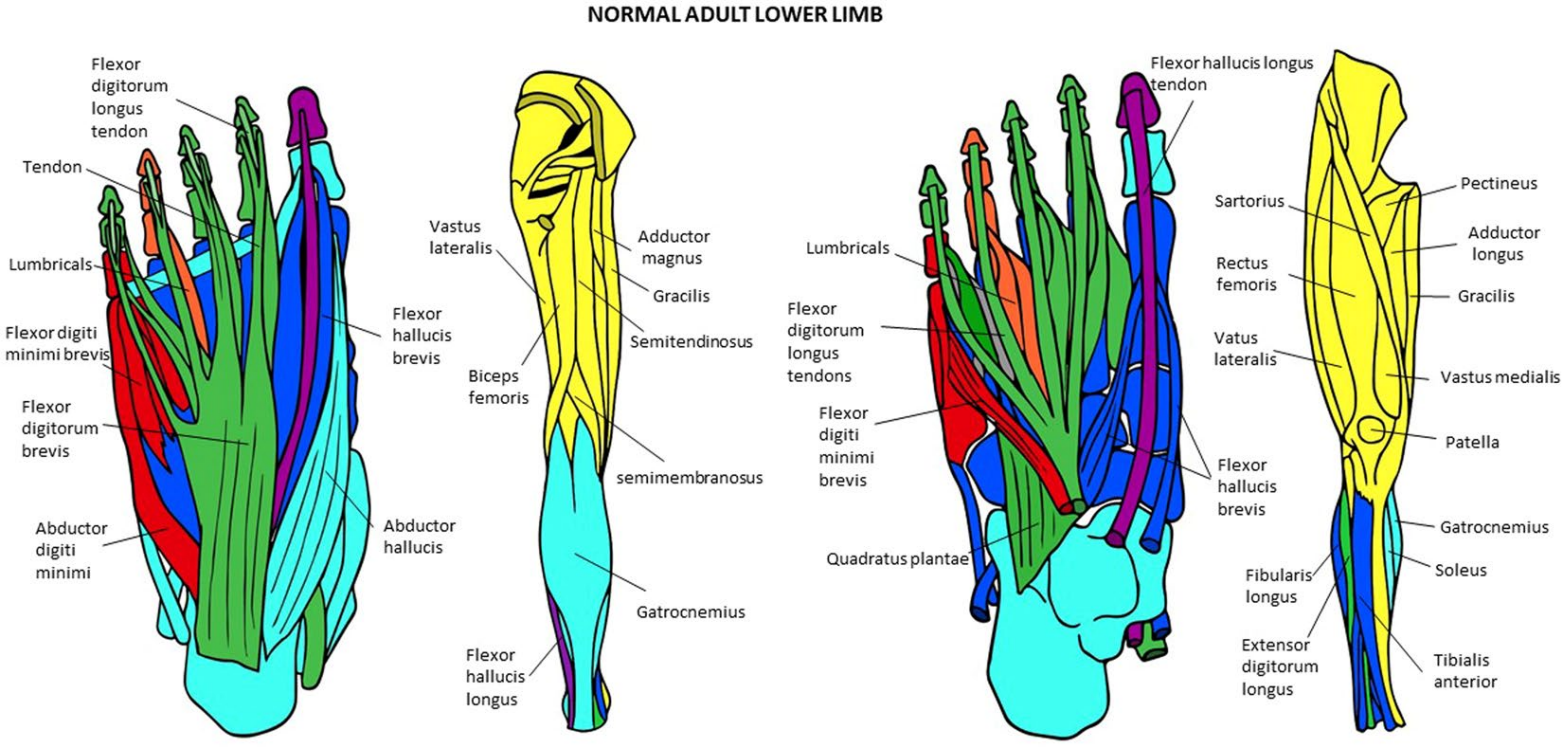
Musculoskeletal modules of the normal adult hindlimb identified using AnNA. Superficial ventral view of foot the left side, then posterior view of thigh and leg, then deep ventral view of foot, and then more to the right anterior view of thigh and leg. In *green*, the module 1 of Table 17 (“digits 2, 3, 5”); in *blue*, the module 2 of Table 17 (“tarsals/metatarsals”); in *turquoise*, the module 3 of Table 17 (“tarsals/1st digit”); in *yellow*, the module 4 of Table 17 (“gluteal/thigh”); in *orange*, the module 5 of Table 17 (“4th digit”); in *red*, the module 7 of Table 17 (“5th digit”); in *purple*, the module 8 of Table 17 (“1st digit”) (other modules of Table 17 not show here).

**Figure 11:**
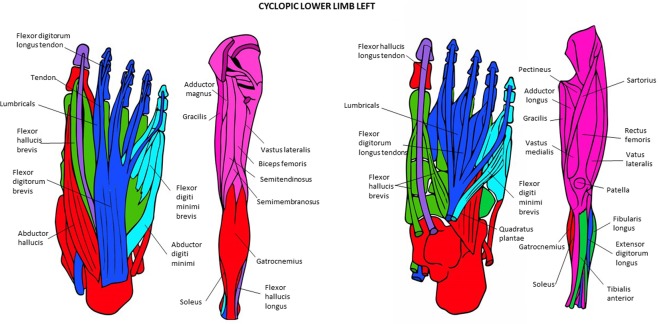
Musculoskeletal modules of the left and right hindlimbs of the T-18 cyclopic fetus identified using AnNA. Superficial ventral view of foot the left side, then posterior view of thigh and leg, then deep ventral view of foot, and then more to the right anterior view of thigh and leg. In *pink*, the module 1 of Tables 22 and 2 of Table 22 (“gluteal/thigh”); in *blue*, the module 2 of Tables 21 and 1 of Table 22 (“2nd, 3rd and 4th digits”); in *green*, the module 3 of Tables 21 and 5 of Table 22 (“tarsals/metatarsals”); in *red*, the module 4 of Tables 21 and 22 (“tarsals/1st digit”); in *turquoise*, the module 5 of Tables 21 and 3 of Table 22 (“5th digit”); in *purple*, the module 7 of Table 21 and Table 22 (“1st digit”) (other modules of Table 21 not show here).

**Figure 12:**
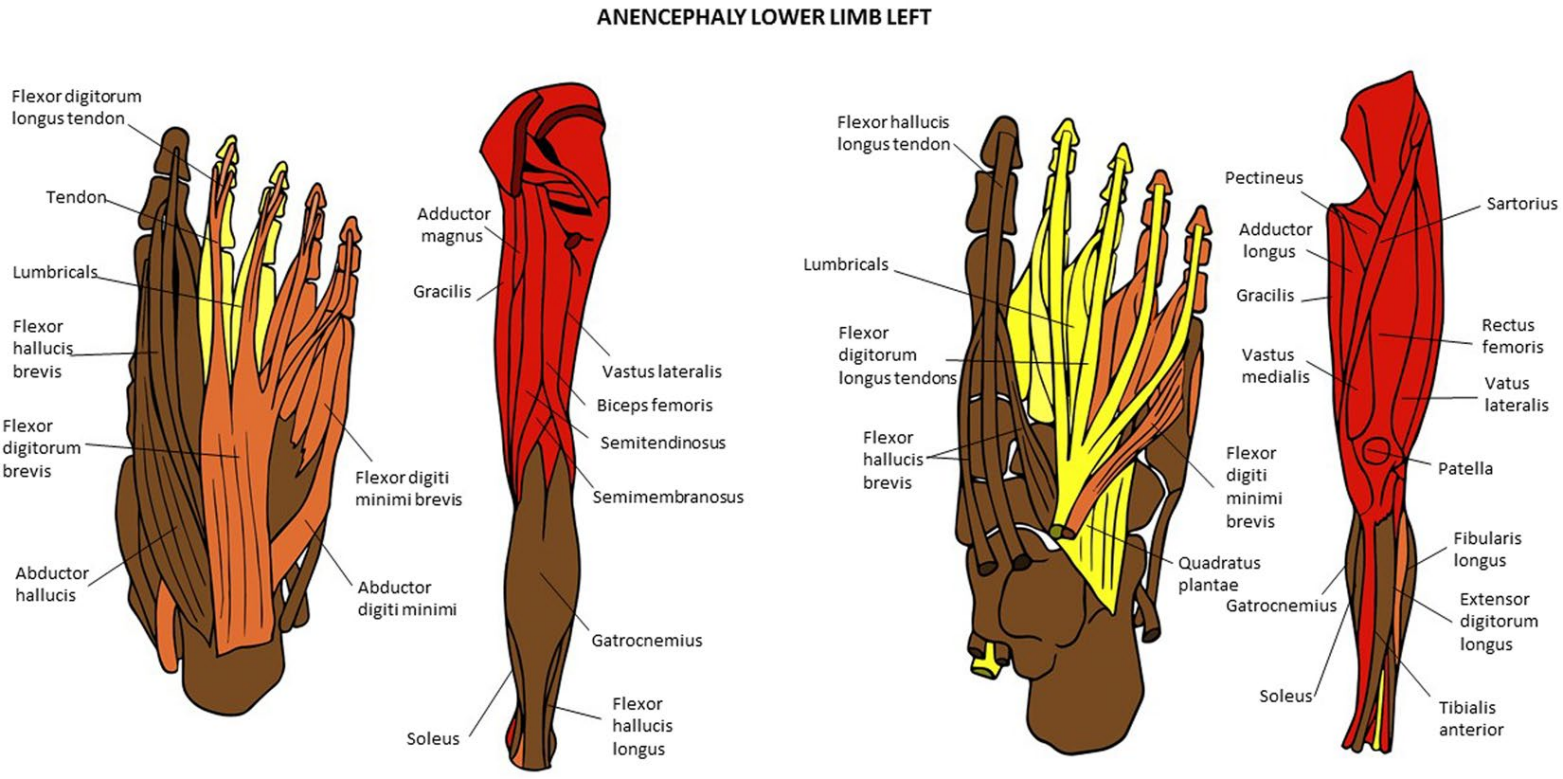
Musculoskeletal modules of the left hindlimb of the anencephaly fetus 1 identified using AnNA. Superficial ventral view of foot the left side, then posterior view of thigh and leg, then deep ventral view of foot, and then more to the right anterior view of thigh and leg. In *brown*, the module 1 of Table 23 (“digit 1/tarsals”); in *red*, the module 2 of Table 23 (“axial/gluteal, thigh”); in *orange*, the module 3 of Table 23 (“digits 4 & 5”); in *yellow*, the module 4 of Table 23 (“digits 2 & 3”).

**Figure 13:**
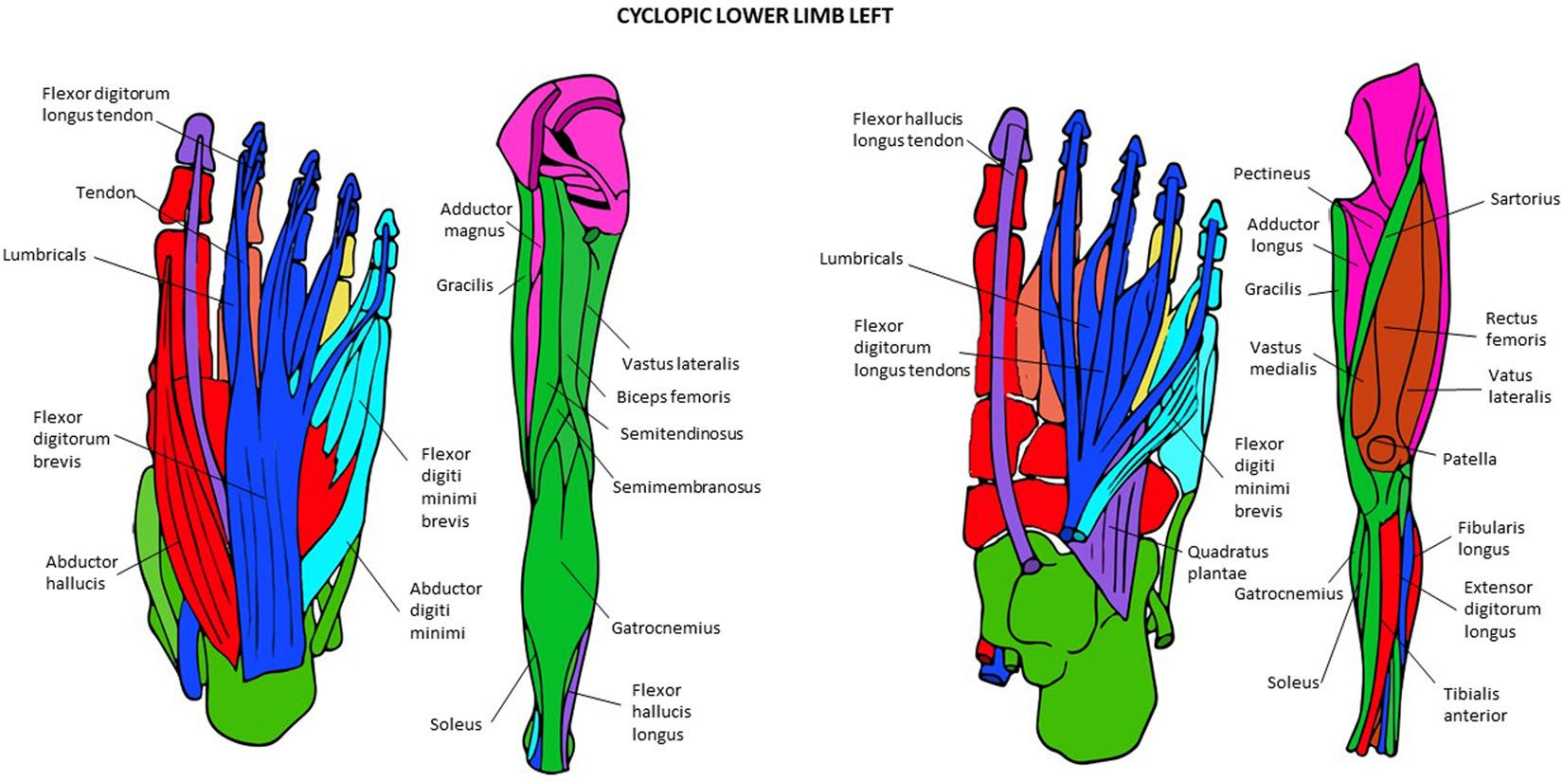
Musculoskeletal modules of the right hindlimb of the anencephaly fetus 1 identified using AnNA. Superficial ventral view of foot the left side, then posterior view of thigh and leg, then deep ventral view of foot, and then more to the right anterior view of thigh and leg. In *brown*, the module 1 of Table 24 (“patella & quadriceps femoris”); in *green*, the module 2 of Table 24 (“thigh”); in *pink*, the module 3 of Table 24 (“gluteal/thigh”); in *turquoise*, the module 4 of Table 24 (“digit 5”); in *red*, the module 6 of Table 24 (“tarsals/digit 1”); in *purple*, the module 7 of Table 24 (“digit 1”); in *blue*, the module 8 of Table 24 (“digits 2, 3, 4”); in *orange*, the module 9 of Table 24 (“digit 2”); in *yellow*, the module 10 of Table 24 (“digit 4”) (other modules of Table 24 not show here).

Here we summarize and compare the AnNA of the five conditions studied: normal adult, normal newborn, normal 7-month fetus, T18 fetus with cyclopia, and anencephaly. The results of the quantification of basic network parameters for the musculoskeletal (including both skeletal and muscular connections) are detailed in Tables 1–24 and Figs 1–13, while all the matrices used for the analyses are detailed in SI. In this paper we focus on the musculoskeletal modular organization of each anatomical system (head, FL, HL) in all the individuals included in the study. In the Discussion section we analyze the broader developmental, evolutionary, and medical implications of these results. It is important to make clear that the names of the modules used in Tables 1–24 and Figs 1–13 are not relevant at all for the network discussions provided in the text. That is, we could actually just call module “A”, or “B”, as we did in some papers, or use names such as “shoulder girdle” or “forearm” to make it a bit less abstract, but of course always having some contradictions. For instance, if in an individual A a “shoulder” module includes 20 structures plus a structure X such as the scapula, and then the exact same 20 structures are present in a module in individual B, but not the scapula, we may still designate that module as “shoulder” in individual B to mean that it has all the same components except that single bone. This is just an informal, not really relevant option, depending on whether we want to create new names for every module, or to try to keep names for mainly similar modules. Even with the use of letters such as A and B, if we would use new names for every single different module, the letters of the alphabet would not be enough, and the paper would be rather chaotic, that is why in all our more recent papers we use names such as “shoulder” or “shoulder and arm” and so on, to try to keep the names as constant as possible, for broader comparisons. What is important is therefore that the readers understand that the names are not important at all, as we are making clear here: what is important is what specific anatomical structures are part of a certain module, as shown specifically in Tables 1–24.

**Table 1:** Musculoskeletal parameters, using AnNA (see text).

Musculoskeletal parameters	N	K	D	C	L	H
Anencephaly 1 head	58	102	0.061706	0.355195	3.804545	0.850846
Anencephaly 2 head	72	172	0.067293	0.47295	3.088811	0.773803
T18 Cyclopic head	94	219	0.050103	0.477792	2.641378	1.228446
Normal 7-month head & neck	120	269	0.037675	0.466439	3.099221	1.088102
Normal newborn head	120	267	0.037395	0.443457	3.10042	1.098521
Normal adult head	117	259	0.038167	0.46701	2.947963	1.197874
Anencephaly 1 left forelimb	92	220	0.052556	0.381752	3.315576	0.686042
Anencephaly 1 right forelimb	64	138	0.068452	0.44762	2.991817	0.713071
T18 Cyclopic left forelimb	90	226	0.056429	0.384506	3.201998	0.694247
T18 Cyclopic right forelimb	87	212	0.056669	0.384841	3.167068	0.740351
Normal 7-month left forelimb	92	230	0.054945	0.405294	3.173913	0.751263
Normal 7-month right forelimb	92	231	0.055184	0.409056	3.173674	0.745664
Normal newborn forelimb	92	222	0.053034	0.371285	3.190874	0.77931
Normal adult forelimb	93	234	0.054698	0.396012	3.187237	0.743795
Anencephaly 1 left hindlimb	93	231	0.053997195	0.371448002	3.351799906	0.695074276
Anencephaly 1 right hindlimb	90	214	0.053433208	0.407361758	3.429213483	0.709996033
T18 Cyclopic left hindlimb	92	225	0.053751	0.37819	3.255375	0.765235
T18 Cyclopic right hindlimb	91	223	0.054457	0.381634	3.263736	0.746928
Normal 7-month left hindlimb	92	228	0.054467	0.389727	3.257525	0.750685
Normal 7-month right hindlimb	92	226	0.053989	0.385997	3.258003	0.761966
Normal newborn hindlimb	92	225	0.053751	0.381601	3.258958	0.767632
Normal adult hindlimb	90	220	0.054931	0.387431	3.158052	0.836054

**Table 2:** Musculoskeletal modularity, using AnNA (see text).

Musculoskeletal modularity	# Modules	Q value	Expected error
Anencephaly 1 head	10	0.45569	0.049859313
Anencephaly 2 head	9	0.482998	0.036962801
T18 Cyclopic head	19	0.382947	0.03657234
Normal 7-month head & neck	7	0.508775	0.028077696
Normal newborn head	9	0.506263	0.029119493
Normal adult head	9	0.463522	0.03146581
Anencephaly 1 left forelimb	7	0.561591	0.028813418
Anencephaly 1 right forelimb	5	0.560098	0.034470211
T18 Cyclopic left forelimb	8	0.541879	0.031696871
T18 Cyclopic right forelimb	9	0.531595	0.0324732
Normal 7-month left forelimb	8	0.54656	0.030912646
Normal 7-month right forelimb	8	0.546532	0.030937066
Normal newborn forelimb	9	0.553608	0.03154872
Normal adult forelimb	9	0.554889	0.030755834
Anencephaly 1 left hindlimb	4	0.562461376	0.024347756
Anencephaly 1 right hindlimb	10	0.531083524	0.032337194
T18 Cyclopic left hindlimb	7	0.545788	0.02745747
T18 Cyclopic right hindlimb	7	0.548583	0.027516686
Normal 7-month left hindlimb	7	0.53982	0.028671697
Normal 7-month right hindlimb	9	0.535447	0.030326795
Normal newborn hindlimb	8	0.541768	0.02909649
Normal adult hindlimb	8	0.535186	0.028776155

**Table 3:** Musculoskeletal modules of the normal adult head using AnNA (see Text).

Module/Complex	p.value	Bones	Muscles
1 **occipital**	0.01092	Occipital, Temporal.Right	Occipitalis, Auricularis.Posterior.Right, Frontalis, Procerus, Stylopharyngeus.Right, Sternocleidomastoideus
2 **hyoid/tongue**	4e-05	Hyoid	Mylohyoideus, Digastricus.Anterior, Stylohyoideus, Digastricus.Posterior, Geniohyoideus, Genioglossus, Hyoglossus, Styloglossus
3 **eye/mastication**	0	Parietals, Temporal.Left, Sphenoid, Zygomatics, Frontal, Mandible	‘eye’: Levator.Palpebrae.Superioris, Superior.Oblique, Superior.Rectus, Inferior.Rectus, Medial.Rectus, Lateral.Rectus, Auricularis.Posterior.Left, Corrugator.Supercilii, Mentalis, ‘mastication’ Masseter, Temporalis.Main.Body, Pterygoideus.Lateralis.Pars.Superior, Pterygoideus.Lateralis.Pars.Inferior, Stylopharyngeus.Left
4 Left & 5 Right **ocular/orofacial**	0.0486		Platysma.Myoides, Risorius, Zygomaticus.Major, Zygomaticus.Minor, Orbicularis.Oculi, Levator.Labii.Superioris.Alaeque.Nasi, Buccinatorius, Levator.Labii.Superioris, Nasalis, Depressor.Septi.Nasi, Levator.Anguli.Oris.Facialis, Orbicularis.Oris, Depressor.Labii.Inferioris, Depressor.Anguli.Oris
6 **eye/nose/mastication**	0.00602	Ethmoid, Nasals, Maxillas, Lacrimals, Palatines, Nasal.Conchas, Vomer	Inferior.Oblique, Depressor.Supercilii, Pterygoideus.Medialis
7 Right & 8 Left **ear**	0.02967		Temporoparietalis, Auricularis.Anterior, Auricularis.Superior
9	1		Trapezius

**Table 4:** Musculoskeletal modules of the normal newborn head using AnNA (see Text).

Module/Complex	p.value	Bones	Muscles
1 **occipital**	0.00457	Occipital, Lateral.Occipitals, Occipital.Plane, Temporal.Left	Occipitalis, Auricularis.Posterior.Left, Stylopharyngeus.Left, Trapezius, Sternocleidomastoideus
2 Right **facial/mastication/tongue**	0.03935	Parietals, Temporal.Right, Zygomatic.Right, Mandible.Right	Auricularis.Posterior.Right, Mentalis.Right, Digastricus.Anterior.Right, Masseter.Right, Temporalis.Main.Body.Right, Pterygoideus.Lateralis.Pars.Superior.Right, Pterygoideus.Lateralis.Pars.Inferior.Right, Stylohyoideus.Right, Digastricus.Posterior.Right, Stylopharyngeus.Right, Hyoglossus.Right, Styloglossus.Right
3 Left **tongue**	0.00124	Mandible.Left, Hyoid	Mentalis.Left, Mylohyoideus, Digastricus.Anterior.Left, Stylohyoideus.Left, Digastricus.Posterior.Left, Geniohyoideus, Genioglossus, Hyoglossus.Left, Styloglossus.Left
4 Left **eye/facial/oral/mastication**	4e-05	Zygomatic.Left, Frontal.Left, Nasal.Left, Maxilla.Left, Lacrimal.Left, Nasal.Concha.Left	Inferior.Oblique.Left, Platysma.Myoides.Left, Risorius.Left, Zygomaticus.Major.Left, Zygomaticus.Minor.Left, Orbicularis.Oculi.Left, Depressor.Supercilii.Left, Corrugator.Supercilii.Left, Levator.Labii.Superioris.Alaeque.Nasi.Left, Buccinatorius.Left, Levator.Labii.Superioris.Left, Nasalis.Left, Depressor.Septi.Nasi.Left, Levator.Anguli.Oris.Facialis.Left, Orbicularis.Oris.Left, Depressor.Labii.Inferioris.Left, Depressor.Anguli.Oris.Left, Masseter.Left, Temporalis.Main.Body.Left
5 Right **eye/facial/oral**	0.00062	Nasal.Right, Maxilla.Right	Inferior.Oblique.Right, Platysma.Myoides.Right, Risorius.Right, Zygomaticus.Major.Right, Depressor.Supercilii.Right, Levator.Labii.Superioris.Alaeque.Nasi.Right, Buccinatorius.Right, Levator.Labii.Superioris.Right, Nasalis.Right, Depressor.Septi.Nasi.Right, Levator.Anguli.Oris.Facialis.Right, Orbicularis.Oris.Right, Depressor.Labii.Inferioris.Right, Depressor.Anguli.Oris.Right
6 **eye/mastication**	0.00146	Sphenoid, Frontal.Right, Ethmoid, Lacrimal.Right, Palatines, Nasal.Concha.Right, Vomer	Levator.Palpebrae.Superioris, Superior.Oblique, Superior.Rectus, Inferior.Rectus, Medial.Rectus, Lateral.Rectus, Zygomaticus.Minor.Right, Orbicularis.Oculi.Right, Corrugator.Supercilii.Right, Pterygoideus.Lateralis.Pars.Superior.Left, Pterygoideus.Lateralis.Pars.Inferior.Left, Pterygoideus.Medialis
7	0.09068		Frontalis, Procerus
8 Left & 9 Right	0.09697		Temporoparietalis, Auricularis.Superior

**Table 5:** Musculoskeletal modules of the normal 7-month old fetus head using AnNA (see Text).

Module/Complex	p.value	Bones	Muscles
1 **occipital**	0.00015	Occipital, Lateral.Occipitals, Occipital.Plane, Parietals, Temporal.Left	Occipitalis, Auricularis.Posterior.Left, Frontalis, Procerus, Masseter.Left, Temporalis.Main.Body.Left, Stylopharyngeus.Left, Trapezius, Sternocleidomastoideus
2 **mastication/tongue**	0	Temporal.Right, Mandibles, Hyoid	Auricularis.Posterior.Right, Mentalis, Mylohyoideus, Digastricus.Anterior, Masseter.Right, Temporalis.Main.Body.Right, Pterygoideus.Lateralis.Pars.Superior.Right, Pterygoideus.Lateralis.Pars.Inferior.Right, Stylohyoideus, Digastricus.Posterior, Stylopharyngeus.Right, Geniohyoideus, Genioglossus, Hyoglossus, Styloglossus
3 Left **eye/facial/oral**	3e-05	Zygomatic.Left, Frontal.Left, Nasal.Left, Maxilla.Left, Lacrimal.Left, Nasal.Concha.Left	Inferior.Oblique.Left, Platysma.Myoides.Left, Risorius.Left, Zygomaticus.Major.Left, Zygomaticus.Minor.Left, Orbicularis.Oculi.Left, Depressor.Supercilii.Left, Corrugator.Supercilii.Left, Levator.Labii.Superioris.Alaeque.Nasi.Left, Buccinatorius.Left, Levator.Labii.Superioris.Left, Nasalis.Left, Depressor.Septi.Nasi.Left, Levator.Anguli.Oris.Facialis.Left, Orbicularis.Oris.Left, Depressor.Labii.Inferioris.Left, Depressor.Anguli.Oris.Left
4 eye/**mastication**	0.00013	Sphenoid, Zygomatic.Right, Frontal.Right, Ethmoid, Nasal.Right, Maxilla.Right, Lacrimal.Right, Palatines, Nasal.Concha.Right, Vomer	Levator.Palpebrae.Superioris, Superior.Oblique, Inferior.Oblique.Right, Superior.Rectus, Inferior.Rectus, Medial.Rectus, Lateral.Rectus, Orbicularis.Oculi.Right, Depressor.Supercilii.Right, Corrugator.Supercilii.Right, Pterygoideus.Lateralis.Pars.Superior.Left, Pterygoideus.Lateralis.Pars.Inferior.Left, Pterygoideus.Medialis
5 Right **facial/oral**	0.01237		Platysma.Myoides.Right, Risorius.Right, Zygomaticus.Major.Right, Zygomaticus.Minor.Right, Levator.Labii.Superioris.Alaeque.Nasi.Right, Buccinatorius.Right, Levator.Labii.Superioris.Right, Nasalis.Right, Depressor.Septi.Nasi.Right, Levator.Anguli.Oris.Facialis.Right, Orbicularis.Oris.Right, Depressor.Labii.Inferioris.Right, Depressor.Anguli.Oris.Right
6 Left & 7 Right	0.09697		Temporoparietalis, Auricularis.Superior

**Table 6:** Musculoskeletal modules of the T-18 cyclopic fetus head using AnNA (see Text).

Module/Complex	p.value	Bones	Muscles
1 **Eye-Mastication**	0.00418	Fused.Central.Bone, Mandible, Sphenoid	Superior.Rectus, Inferior.Rectus.Single, Mentalis, Pterygoideus.Lateralis.Pars.Superior, Pterygoideus.Lateralis.Pars.Inferior, Pterygoideus.Medialis
2 **Skull-mastication-facial**	0.00355	Occipital, Lateral.Occipitals, Parietals, Temporals, Zygomatics, Frontal	Zygomaticus.Minor, Orbicularis.Oculi, Masseter, Temporalis.Main.Body
3 Left & 4 Right **Facial**	0.00481		Platysma.Myoides, Risorius, Zygomaticus.Major, Levator.Labii.Superioris.Alaeque.Nasi, Buccinatorius, Levator.Labii.Superioris, Nasalis, Depressor.Septi.Nasi, Levator.Anguli.Oris.Facialis, Orbicularis.Oris, Depressor.Labii.Inferioris, Depressor.Anguli.Oris
5	0.09068		Occipitalis, Frontalis
6	0.0694	Occipital.Plane	Trapezius, Sternocleidomastoideus
7	0.116	Hyoid	Geniohyoideus, Genioglossus
8 Right & 9 Left	1		Hyoglossus, Styloglossus
10	0.09068		Mylohyoideus, Digastricus.Anterior
11	0.56567		Inferior.Oblique, Lateral.Rectus
12 Right R & 13 Left	0.93319		Stylohyoideus, Digastricus.Posterior
14 Right & 15 Left	0.09697		Temporoparietalis, Auricularis.Superior
16	1		Auricularis.Posterior.Left
17	1		Auricularis.Posterior.Right
18	1		Stylopharyngeus.Left
19	1		Stylopharyngeus.Right

**Table 7:** Musculoskeletal modules of the anencephalic fetus 1 head using AnNA (see Text).

Module/Complex	p.value	Bones	Muscles
1 Left **upper-mid face**	0.00688	Frontal.Left, Zygomatic.Left, Maxilla.Left, Interparietal.Left, Exooccipital.Left, Sphenoid.Left	Frontalis.Left, Auricularis.Superior.Left, Orbicularis.Oculi.Left, Levator.Labii.Superioris.Left, Zygomaticus.Major.Left, Zygomaticus.Minor.Left, Temporalis.Left
2 Left **skull-mandible-lower face**	0.3549	Mandible.Left, Petrous.Temporals,	Auricularis.Posterior.Left, Orbicularis.Oris, Depressor.Labii.Inferioris.Left, Mentalis.Left, Buccinator.Left, Masseter.Left
3 Right **mandible-lower face**	0.02036	Mandible.Right	Depressor.Anguli.Oris.Right, Depressor.Labii.Inferioris.Right, Mentalis.Right, Mylohyoideus, Digastricus.Anterior
4 Right **upper-mid face**	0.00669	Frontal.Right, Zygomatic.Right, Maxilla.Right, Nasal, Vomer, Ethmoid, Sphenoid.Right	Frontalis.Right, Orbicularis.Oculi.Right, Procerus, Nasalis.Left
5 **skull-digastric-neck muscles**	0.00376	Supraoccipital.Right., Interparietal.Right, Exooccipital.Right,	Digastricus.Posterior.Right, Trapezius.Right, Sternocleidomastoideus.Right
6	0.40683		Risorius.Left, Depressor.Anguli.Oris.Left, Platysma
7	0.08602	Supraoccipital.Left,	Digastricus.Posterior.Left, Trapezius.Left, Sternocleidomastoideus.Left
8	1		Extra.Muscle.Over.Nose.Right, Strange.Muscle.Over.Nose.Left
9	1		Buccinator.Right
10	1		Masseter.Right

**Table 8:** Musculoskeletal modules of the anencephalic fetus 2 head using AnNA (see Text).

Module/Complex	p.value	Bones	Muscles
1 **hyoid and tongue**	0.00127	Hyoid	Mylohyoideus, Geniohyoideus, Genioglossus, Hyoglossus
2 **face and neurocranium**	0.01104	Frontals, Nasal (single), Vomer, Exooccipital.Left, Basioccipital, Parietals, Sphenoids, Ethmoid	Procerus, Nasalis
3 Right **face and facial muscles**	0.00437	Zygomatic.Right, Maxilla.Right, Mandible.Right	Orbicularis.Oculi.Right, Levator.Labii.Superioris.Right, Zygomaticus.Major.Right, Zygomaticus.Minor.Right, Levator.Labii.Superioris.Alaeque.Nasi.Right, Levator.Anguli.Oris.Right, Depressor.Anguli.Oris.Right, Buccinator.Right, Masseter.Right, Digastricus.Anterior.Right
4 Left **face and facial muscles**	0.00721	Zygomatic.Left, Maxilla.Left, Mandible.Left,	Orbicularis.Oculi.Left, Orbicularis.Oris, Levator.Labii.Superioris.Left, Zygomaticus.Major.Left, Zygomaticus.Minor.Left, Levator.Labii.Superioris.Alaeque.Nasi.Left, Levator.Anguli.Oris.Left, Buccinator.Left, Masseter.Left, Digastricus.Anterior.Left
5 Right **cranium and trapezius**	0.40683	Supraoccipital.Interparietal.Right, Exooccipital.Right	Trapezius.Right
6 Left and 7 Right **temporal and neck muscles**	0.19443	Temporal	Temporalis, Digastricus.Posterior, Stylohyoideus, Sternocleidomastoideus, Styloglossus
8 Left **cranium and trapezius**	0.65845	Supraoccipital.Interparietal.Left	Trapezius.Left
9	0.05565		Depressor.Anguli.Oris.Left, Depressor.Labii.Inferioris, Mentalis, Platysma

**Table 9:** Musculoskeletal modules of normal adult forelimb using AnNA (see Text). ant., anterior; comp., compartment; post., posterior.

ID	p.value	Bones	Muscles
1 **shoulder girdle**	0.00183	Vertebrae, Ribs, Sternum, Clavicle	**chest:** Subclavius, Pectoralis.Major, Pectoralis.Minor, **scapula:** Deltoid, Serratus.Anterior, Levator.Scapulae, Rhomboid.Minor, Rhomboid.Major, Latissimus.Dorsi
2 **arm, forearm**	9e-05	Scapula, Humerus, Radius, Ulna	**scapula:** Supraspinatus, Infraspinatus, Teres.Minor, Teres.Major, Subscapularis, **ant. comp. arm:** Biceps.Brachii, Coracobrachialis, Brachialis, **post. comp. arm:** Triceps.Brachii, **post. comp. forearm:** Anconeus, Brachioradialis, Extensor.Carpi.Ulnaris, Supinator, Abductor.Pollicis.Longus, **ant. comp. forearm:** Pronator.Teres, Palmaris.Longus, Flexor.Digitorum.Superficialis, Pronator.Quadratus
3 **digit 3**	0.00312	Metacarpals 2,3, Proximal.Phalanx.3, Middle.Phalanx.3, Distal.Phalanx.3	**ant. comp. forearm:** Flexor.Carpi.Radialis, **post. comp. forearm:** Extensor.Carpi.Radialis.Longus, Extensor.Carpi.Radialis.Brevis, **hand:** Lumbrical.2, Dorsal.Interossei 2,3
4 **carpals, digit 5**	0.17434	Lunate, Triquetrum, Pisiform, Hamate, Metacarpal.5	Flexor.Carpi.Ulnaris, Opponens.Digiti.Minimi, Abductor.Digiti.Minimi
5 **digit 5**	0.0486	Proximal.Phalanx.5, Middle.Phalanx.5, Distal.Phalanx.5	**ant. comp. forearm:** Flexor.Digitorum.Profundus, **post. comp. forearm**: Extensor.Digitorum, Extensor.Digiti.Minimi, **hand**: Flexor.Digiti.Minimi.Brevis, Lumbrical.4, Palmar.Interosseus 3
6 **carpals, digit 1**	0.00027	Trapezoid, Trapezium, Scaphoid, Capitate, Metacarpal.1, Proximal.Phalanx.1	**post. comp. forearm:** Extensor.Pollicis.Brevis, Abductor.Pollicis.Brevis, **thenar comp.:** Adductor.Pollicis, Adductor.Pollicis.Accessorius, Flexor.Brevis.Profundus.2, Flexor.Pollicis.Brevis, Opponens.Pollicis
7 **digit 4**	0.00988	Metacarpal.4, Proximal.Phalanx.4, Middle.Phalanx.4, Distal.Phalanx.4	Lumbrical.3, Dorsal.Interosseus 4, Palmar.Interosseus 2
8 **digit 2**	0.00157	Proximal.Phalanx.2, Middle.Phalanx.2, Distal.Phalanx.2	Extensor.Indicis, Lumbrical.1, Dorsal.Interosseus 1, Palmar.Interosseus 1
9 **distal phalanx 1**	0.25249	Distal.Phalanx.1	Flexor.Pollicis.Longus, Extensor.Pollicis.Longus

**Table 10:** Musculoskeletal modules of normal newborn forelimb using AnNA (see Text). ant., anterior; comp., compartment; post., posterior.

ID	p.value	Bones	Muscles
1 **shoulder girdle**	0.00329	Vertebrae, Ribs, Sternum, Clavicle	**chest**: Subclavius, Pectoralis.Major, Pectoralis.Minor, **scapula**: Serratus.Anterior, Levator.Scapulae, Rhomboid.Minor, Rhomboid.Major, Latissimus.Dorsi
2 **arm, forearm**	7e-05	Scapula, Humerus, Radius, Ulna	**scapula**: Deltoid, Supraspinatus, Infraspinatus, Teres.Minor, Teres.Major, Subscapularis, a**nt. comp. arm:** Biceps.Brachii, Coracobrachialis, Brachialis, post. comp. arm: Triceps.Brachii, **post. comp. forearm:** Anconeus, Brachioradialis, Extensor.Carpi.Ulnaris, Supinator, Abductor.Pollicis.Longus, **ant. comp. forearm:** Pronator.Teres, Palmaris.Longus, Flexor.Digitorum.Superficialis, Pronator.Quadratus
3 **carpals, digit 5**	0.10569	Lunate, Triquetrum, Pisiform, Hamate, Metacarpal.5	Flexor.Carpi.Ulnaris, Opponens.Digiti.Minimi, Abductor.Digiti.Minimi
4 **digit 3**	0.00869	Metacarpals 2,3, Proximal.Phalanx.3, Middle.Phalanx.3, Distal.Phalanx.3	**ant. comp. forearm**: Flexor.Carpi.Radialis, **post. comp. forearm:** Extensor.Carpi.Radialis.Longus, Extensor.Carpi.Radialis.Brevis, **hand:** Lumbrical.2, Dorsal.Interossei 2,3
5 **digit 5**	0.05784	Proximal.Phalanx.5, Middle.Phalanx.5, Distal.Phalanx.5	Flexor.Digitorum.Profundus, Extensor.Digitorum, Extensor.Digiti.Minimi, Flexor.Digiti.Minimi.Brevis, Lumbrical.4, Palmar.Interosseus 3
6 **carpals, digit 1**	0.00064	Trapezoid, Trapezium, Scaphoid, Capitate, Metacarpal.1, Proximal.Phalanx.1	Extensor.Pollicis.Brevis, Adductor.Pollicis, Adductor.Pollicis.Accessorius, Abductor.Pollicis.Brevis, Flexor.Pollicis.Brevis, Opponens.Pollicis
7 **digit 4**	0.00988	Metacarpal.4, Proximal.Phalanx.4, Middle.Phalanx.4, Distal.Phalanx.4	Lumbrical.3, Dorsal.Interosseus 4, Palmar.Interosseus 2
8 **digit 2**	0.00157	Proximal.Phalanx.2, Middle.Phalanx.2, Distal.Phalanx.2	Extensor.Indicis, Lumbrical.1, Dorsal.Interosseus 1, Palmar.Interosseus 1
9 **distal phalanx 1**	0.25249	Distal.Phalanx.1	Flexor.Pollicis.Longus, Extensor.Pollicis.Longus

**Table 11:** Musculoskeletal modules of left forelimb of normal 7-month fetus using AnNA (see Text). ant., anterior; comp., compartment; post., posterior.

ID	p.value	Bones	Muscles
1 **shoulder girdle**	0.00082	Vertebrae, Ribs, Sternum, Clavicle	**chest**: Subclavius, Pectoralis.Major, Pectoralis.Minor, **scapula**: Serratus.Anterior, Levator.Scapulae, Rhomboid.Minor, Rhomboid.Major, Latissimus.Dorsi
2 **carpals, digit 5**	0.00141	Lunate, Triquetrum, Pisiform, Hamate, Metacarpal.5, Proximal.Phalanx.5, Middle.Phalanx.5, Distal.Phalanx.5	**ant. comp. forearm**: Flexor.Carpi.Ulnaris, Flexor.Digitorum.Profundus, **post. comp. forearm**: Extensor.Digitorum, Extensor.Digiti.Minimi, Abductor.Digiti.Minimi, **hand**: Opponens.Digiti.Minimi, Flexor.Digiti.Minimi.Brevis, Lumbrical.4, Palmar.Interosseus 3
3 **arm, forearm**	5e-05	Scapula, Humerus, Radius, Ulna	**scapula**: Deltoid, Supraspinatus, Infraspinatus, Teres.Minor, Teres.Major, Subscapularis, **ant. comp. arm**: Biceps.Brachii, Coracobrachialis, Brachialis, post. comp. arm: Triceps.Brachii, **post. comp. forearm**: Anconeus, Brachioradialis, Extensor.Carpi.Ulnaris, Supinator, Abductor.Pollicis.Longus, **ant. comp. forearm**: Pronator.Teres, Palmaris.Longus, Flexor.Digitorum.Superficialis, Pronator.Quadratus
4 **digit 3**	0.00483	Capitate, Metacarpals 2,3, Proximal.Phalanx.3, Middle.Phalanx.3, Distal.Phalanx.3	**ant. comp. forearm**: Flexor.Carpi.Radialis, **post. comp. forearm**: Extensor.Carpi.Radialis.Longus, Extensor.Carpi.Radialis.Brevis, **hand**: Adductor.Pollicis, Lumbrical.2, Dorsal.Interossei 2,3
5 **carpals, digit 1**	0.00513	Trapezoid, Trapezium, Scaphoid, Metacarpal.1, Proximal.Phalanx.1	Extensor.Pollicis.Brevis, Abductor.Pollicis.Brevis, Adductor.Pollicis.Accessorius, Flexor.Pollicis.Brevis, Opponens.Pollicis
6 **digit 4**	0.00988	Metacarpal.4, Proximal.Phalanx.4, Middle.Phalanx.4, Distal.Phalanx.4	Lumbrical.3, Dorsal.Interosseus 4, Palmar.Interosseus 2
7 **digit 2**	0.00157	Proximal.Phalanx.2, Middle.Phalanx.2, Distal.Phalanx.2,	Extensor.Indicis, Lumbrical.1, Dorsal.Interosseus 1, Palmar.Interosseus 1
8 **distal phalanx 1**	0.25249	Distal.Phalanx.1	Flexor.Pollicis.Longus, Extensor.Pollicis.Longus

**Table 12:** Musculoskeletal modules of right forelimb of normal 7-month fetus using AnNA (see Text). ant., anterior; comp., compartment; post., posterior.

ID	p.value	Bones	Muscles
1 **shoulder girdle**	0.00082	Vertebrae, Ribs, Sternum, Clavicle	**chest**: Subclavius, Pectoralis.Major, Pectoralis.Minor, **scapula**: Serratus.Anterior, Levator.Scapulae, Rhomboid.Minor, Rhomboid.Major, **back**: Latissimus.Dorsi
2 **carpals, digit 5**	0.00141	Lunate, Triquetrum, Pisiform, Hamate, Metacarpal.5, Proximal.Phalanx.5, Middle.Phalanx.5, Distal.Phalanx.5	**ant. comp. forearm**: Flexor.Carpi.Ulnaris, Flexor.Digitorum.Profundus, **post. comp. forearm**: Extensor.Digitorum, Extensor.Digiti.Minimi, Abductor.Digiti.Minimi, **minimi**: Opponens.Digiti.Minimi, Flexor.Digiti.Minimi.Brevis, Lumbrical.4, Palmar.Interosseus 3
3 **arm, forearm**	0.00012	Scapula, Humerus, Radius, Ulna	**scapula**: Deltoid, Supraspinatus, Infraspinatus, Teres.Minor, Teres.Major, Subscapularis, **ant. comp. arm**: Biceps.Brachii, Coracobrachialis, Brachialis, **post. comp. arm**: Triceps.Brachii, **post. comp. forearm**: Anconeus, Brachioradialis, Extensor.Carpi.Ulnaris, Supinator, **ant. comp. forearm**: Pronator.Teres, Palmaris.Longus, Flexor.Digitorum.Superficialis, Pronator.Quadratus
4 **digit 3**	0.00483	Capitate, Metacarpals 2,3, Proximal.Phalanx.3, Middle.Phalanx.3, Distal.Phalanx.3	**ant. comp. forearm**: Flexor.Carpi.Radialis, **post. comp. forearm**: Extensor.Carpi.Radialis.Longus, Extensor.Carpi.Radialis.Brevis, **hand**: Adductor.Pollicis, Lumbrical.2, Dorsal.Interossei 2,3
5 **carpals, digit 1**	0.00131	Trapezoid, Trapezium, Scaphoid, Metacarpal.1, Proximal.Phalanx.1	**post. comp. forearm**: Abductor.Pollicis.Longus, Abductor.Pollicis.Brevis, Extensor.Pollicis.Brevis, **thenar comp**.: Adductor.Pollicis.Accessorius, Flexor.Pollicis.Brevis, Opponens.Pollicis
6 **digit 4**	0.00988	Metacarpal.4, Proximal.Phalanx.4, Middle.Phalanx.4, Distal.Phalanx.4	Lumbrical.3, Dorsal.Interosseus 4, Palmar.Interosseus 2
7 **digit 2**	0.00157	Proximal.Phalanx.2, Middle.Phalanx.2, Distal.Phalanx.2,	Extensor.Indicis, Lumbrical.1, Dorsal.Interosseus 1, Palmar.Interosseus 1
8 **distal phalanx 1**	0.25249	Distal.Phalanx.1	Flexor.Pollicis.Longus, Extensor.Pollicis.Longus

**Table 13:** Musculoskeletal modules of left forelimb of T18 cyclopic fetus using AnNA (see Text). ant., anterior; comp., compartment; post., posterior.

ID	p.value	Bones	Muscles
1 **carpals, digit 5**	0.00011	Lunate, Triquetrum, Pisiform, Hamate, Metacarpal.5, Proximal.Phalanx.5, Middle.Phalanx.5, Distal.Phalanx.5	Flexor.Carpi.Ulnaris, Abductor.Digiti.Minimi, Lumbrical.4, Palmar.Interosseus 3, **hypothenar**: Opponens.Digiti.Minimi, Flexor.Digiti.Minimi.Brevis
2 **shoulder girdle A**	0.0234	Ribs, Sternum, Clavicle	**scapula**: Subclavius, Deltoid, Serratus.Anterior, **chest**: Pectoralis.Major, Pectoralis.Minor, **ant. comp. arm**: Biceps.Brachii
3 **arm, forearm, digit 1**	7e-04	Humerus, Radius, Ulna, Distal.Phalanx.1	**ant. comp. arm**: Brachialis, **post. comp. arm**: Triceps.Brachii, ant. comp. forearm: Pronator.Teres, Flexor.Carpi.Radialis, Flexor.Digitorum.Superficialis, Flexor.Pollicis.Longus, Pronator.Quadratus, **post. comp. forearm**: Anconeus, Brachioradialis, Extensor.Carpi.Radialis.Longus, Extensor.Carpi.Radialis.Brevis, Extensor.Digitorum, Extensor.Digiti.Minimi, Extensor.Carpi.Ulnaris, Supinator, Extensor.Pollicis.Longus
4 **shoulder girdle B**	0.01325	Vertebrae, Scapula	Supraspinatus, Infraspinatus, Teres.Minor, Teres.Major, Subscapularis, Levator.Scapulae, Rhomboid.Major.Minor, Latissimus.Dorsi, Coracobrachialis
5 **carpals, digit 1**	0.00025	Trapezoid, Trapezium, Scaphoid, Capitate, Metacarpals 1,2,3, Proximal.Phalanx.1	**post**.: Abductor.Pollicis.Longus, Abductor.Pollicis.Brevis, Extensor.Pollicis.Brevis, **ant**: Adductor.Pollicis, Adductor.Pollicis.Accessorius, Musculus.Interosseous.Accessorius, Flexor.Pollicis.Brevis, Opponens.Pollicis
6 **digit 3**	0.02213	Proximal.Phalanx.3, Middle.Phalanx.3, Distal.Phalanx.3	Flexor.Digitorum.Profundus, Lumbrical.2, Dorsal.Interossei.2,3
7 **digit 4**	0.00988	Metacarpal.4, Proximal.Phalanx.4, Middle.Phalanx.4, Distal.Phalanx.4,	Lumbrical.3, Dorsal.Interosseus 4, Palmar.Interosseus 2
8 **digit 2**	0.00469	Proximal.Phalanx.2, Middle.Phalanx.2, Distal.Phalanx.2	Extensor.Indicis, Dorsal.Interosseus 1, Palmar.Interosseus 1

**Table 14:** Musculoskeletal modules of right forelimb of T18 cyclopic fetus using AnNA (see Text). ant., anterior; comp., compartment; post., posterior.

ID	p.value	Bones	Muscles
1 **shoulder girdle**	0.02053	Ribs, Sternum, Clavicle	Subclavius, Deltoid, Pectoralis.Major, Pectoralis.Minor, Serratus.Anterior
2 **digits 2 & 3**	1e-04	Proximal.Phalanges 2,3, Middle.Phalanges 2,3, Distal.Phalanges 2,3	**ant. comp. forearm**: Flexor.Digitorum.Superficialis, Flexor.Digitorum.Profundus, **post. comp. forearm**: Extensor.Digitorum, Extensor.Indicis, **hand**: Musculus.Interosseous.Accessorius, Lumbrical.2, Dorsal.Interossei 1,2,3, Palmar.Interosseus 1
3 **arm, forearm**	0.00011	Scapula, Humerus, Radius, Ulna	**scapula**: Supraspinatus, Infraspinatus, Teres.Minor, Subscapularis, **ant. comp. arm**: Biceps.Brachii, Coracobrachialis, Brachialis, **post. comp. arm**: Triceps.Brachii, **ant. comp. forearm**: Pronator.Teres, Palmaris.Longus, Pronator.Quadratus, **post. comp. forearm**: Anconeus, Brachioradialis, Extensor.Carpi.Ulnaris, Supinator, Abductor.Pollicis.Longus
4 **carpals, digit 5**	0.03844	Triquetrum, Pisiform, Hamate, Metacarpal.5, Proximal.Phalanx.5	Flexor.Carpi.Ulnaris, **hypothenar comp**.: Opponens.Digiti.Minimi, Flexor.Digiti.Minimi.Brevis, **dorsal**: Abductor.Digiti.Minimi
5 **digit 1**	0.33219	Metacarpal.1, Proximal.Phalanx.1, Distal.Phalanx.1	Extensor.Pollicis.Longus, Adductor.Pollicis.Accessorius
6 **carpals, metacarpals**	0.02119	Trapezoid, Trapezium, Scaphoid, Lunate, Capitate, Metacarpals 2,3	**ant. comp. forearm**: Flexor.Carpi.Radialis, Flexor.Pollicis.Longus, **post. comp. forearm**: Extensor.Carpi.Radialis.Longus, Extensor.Carpi.Radialis.Brevis, **thenar m**.: Adductor.Pollicis
7 posterior shoulder	0.2593	Vertebrae	Teres.Major, Levator.Scapulae, Rhomboid.Major.Minor, Latissimus.Dorsi
8 digit 4	0.00988	Metacarpal.4, Proximal.Phalanx.4, Middle.Phalanx.4, Distal.Phalanx.4	Lumbrical.3, Dorsal.Interosseus 4, Palmar.Interosseus 2
9 digit 5	0.02063	Middle.Phalanx.5, Distal.Phalanx.5	Extensor.Digiti.Minimi, Lumbrical.4, Palmar.Interosseus 3

**Table 15:** Musculoskeletal modules of left forelimb of anencephalic fetus 1 using AnNA (see Text). ant., anterior; comp., compartment; post., posterior.

ID	p.value	Bones	Muscles
1 **carpals, digit 1**	2e-05	Scaphoid, Lunate, Triquetrum, Trapezium, Trapezoid, Capitate, Metacarpals 1,2,3 Proximal.Phalanx.1, Distal.Phalanx.1	**ant. comp. forearm**: Flexor.Carpi.Radialis, Flexor.Pollicis.Longus, Palmaris.Brevis, **post. comp. forearm**: Brachioradialis, Extensor.Carpi.Radialis.Longus, Extensor.Carpi.Radialis.Brevis, Extensor.Pollicis.Longus, **thenar compartment**: Flexor.Pollicis.Brevis, Adductor.Pollicis, Opponens.Pollicis, Flexor.Brevis.Profundus.2, Adductor.Pollicis.Accessorius
2 **shoulder girdle, arm, forearm**	0	Ribs, Sternum, Clavicle., Scapula, Humerus, Radius, Ulna	**chest**: Pectoralis.Major, Pectoralis.Minor, **scapula**: Deltoid, Supraspinatus, Infraspinatus, Teres.Minor, Teres.Major, **ant. comp. arm**: Biceps.Brachii, Coracobrachialis, Brachialis, **post. comp. arm**: Triceps.Brachii, **ant. comp. forearm**: Pronator.Teres, Flexor.Carpi.Ulnaris, Flexor.Digitorum.Superficialis, Pronator.Quadratus, **post. comp. forearm**: Extensor.Carpi.Ulnaris, Anconeus, Supinator, **other**: Latissimus.Dorsi, Extra.Muscle
3 **carpals, digit 5**	0.00112	Pisiform, Hamate, Metacarpal.5, Proximal.Phalanx.5, Middle.Phalanx.5, Distal.Phalanx.5	**dorsal**: Extensor.Digiti.Minimi, Abductor.Digiti.Minimi, **hypothenar mm**.: Opponens.Digiti.Minimi, Flexor.Digiti.Minimi.Brevis, **others**: Lumbrical.4, Palmar.Interosseus 3
4 **digits 3 & 4**	2e-04	Metacarpal.4, Proximal.Phalanges 3,4, Middle.Phalanges 3,4, Distal.Phalanges 3,4	Flexor.Digitorum.Profundus, Extensor.Digitorum, Lumbricals 2,3, Palmar.Interosseus 2, Dorsal.Interossei 2,3,4
5 **posterior shoulder**	0.03732	Occipital bone, Vertebrae	Rhomboideus.Occipitalis, Rhomboid.Major, Rhomboid.Minor
6 **pollicis**	0.15085		Abductor.Pollicis.Longus, Abductor.Pollicis.Brevis, Extensor.Pollicis.Brevis
7 **digit 2**	0.00168	Proximal.Phalanx.2, Middle.Phalanx.2, Distal.Phalanx.2	Extensor.Indicis, Lumbrical.1, Palmar.Interosseous.1, Dorsal.Interosseous.1

**Table 16:** Musculoskeletal modules of right forelimb of anencephalic fetus 1 using AnNA (see Text). ant., anterior; comp., compartment; post., posterior.

ID	p.value	Bones	Muscles
1 **carpals, digit 3**	5e-05	Ulna, Triquetrum, Metacarpal.3, Proximal.Phalanx.3, Middle.Phalanx.3, Distal.Phalanx.3	**ant. comp. forearm**: Flexor.Carpi.Ulnaris, Palmaris.Longus, Flexor.Digitorum.Superficialis, Flexor.Digitorum.Profundus, **post.comp. forearm**: Extensor.Digitorum, Extensor.Carpi.Ulnaris, Extensor.Pollicis.Longus, Extensor.Indicis, Abductor.Pollicis.Longus, Anconeus, **intrinsic hand**: Dorsal.Interossei 1, 2, **others**: Anomaly.1, Anomaly.2
2 **shoulder girdle, arm, forearm**	0	Vertebrae, Ribs, Sternum, Clavicle, Scapula, Humerus	**chest**: Pectoralis.Major, Pectoralis.Minor, **scapula**: Deltoid, Supraspinatus, Infraspinatus., Teres.Minor, Teres.Major, Rhomboid.Major, Rhomboid.Minor, **back**: Latissimus.Dorsi, Dorsoepitrochlearis, **ant. comp. arm**: Biceps.Brachii.Short.Head, Coracobrachialis, **post. comp. arm**: Triceps.Brachii, **ant. comp. forearm**: Pronator.Teres
3 **carpals, digit 5**	4e-05	Pisiform, Hamate, Fused.Carpals, Metacarpal.5, Proximal.Phalanx.5, Middle.Phalanx.5, Distal.Phalanx.5	**hypothenar & 5**^**th**^ **digit**: Extensor.Digiti.Minimi, Abductor.Digiti.Minimi, Opponens.Digiti.Minimi, Flexor.Digiti.Minimi.brevis, Lumbrical.4, other: Palmar.Interosseus.2
4 **digit 1**	0.02967	Metacarpal.1, Proximal.Phalanx.1, Distal.Phalanx.1	
5 **digit 4**	0.00089	Metacarpal.4, Proximal.Phalanx.4, Middle.Phalanx.4, Distal.Phalanx.4	Lumbrical.3, Palmar.Interosseus.1, Dorsal.Interosseus.3

**Table 17:** Musculoskeletal modules of hindlimb of normal adult using AnNA (see Text). ant., anterior; comp., compartment; post., posterior.

ID	p.value	Bones	Muscles
1 **digits 2,3,5**	0	Proximal.Phalanges 2,3, Middle.Phalanges 2,3,5, Distal.Phalanges 2,3,5	**ant. comp. leg**: Extensor.Digitorum.Longus, Fibularis.Tertius, Extensor.Digitorum.Brevis, **deep post. comp. leg**: Flexor.Digitorum.Longus, **foot**: Flexor.Digitorum.Brevis, Quadratus.Plantae, Lumbricals 1,2,4, Plantar.Interosseus.1, Dorsal.Interossei 1,2,3
2 **tarsals, metatarsals**	0.00028	Navicular, Cuboid, Medial.Cuneiform, Intermediate.Cuneiform, Lateral.Cuneiform, Metatarsals 1,2,3,4	Fibularis.Longus, Tibialis.Anterior, Tiblialis.Posterior, Flexor.Hallucis.Brevis, Adductor.Hallucis
3 **tarsals, digit 1**	0.21666	Fibula, Calcaneus, Talus, Proximal.Phalanx.1	Plantaris, Gastrocnemius, Soleus, Fibularis.Brevis, Extensor.Hallucis.Brevis, Abductor.Hallucis
4 **gluteal, thigh**	0	Hip.Bone, Femur, Patella, Tibia	**gluteal**: Gluteus.Medius, Gluteus.Minimus, Tensor.Fasciae.Latae, Obturator.Internus, Obturator.Externus, Gemellus.Superior, Gemellus.Inferior, Quadratus.Femoris, **anterior comp. thigh**: Sartorius, **quadriceps femoris**: Rectus.Femoris, Vastus.Lateralis, Vastus.Intermedius, Vastus.Medialis, **med. comp. thigh**: Pectineus, Adductor.Brevis, Adductor.Magnus, Adductor.Longus, Gracilis **Hamstrings**: Biceps.Femoris, Semitendinosus, Semimembranosus, Popliteus
5 **digit 4**	0.04496	Proximal.Phalanx.4, Middle.Phalanx.4, Distal.Phalanx.4	Lumbrical.3, Plantar.Interosseus.2, Dorsal.Interosseus.4
6 **axial**	0.32565	Vertebrae, Sacrum	Gluteus.Maximus, Piriformis, Iliopsoas
7 **digit 5**	0.11641	Metatarsal.5, Proximal.Phalanx.5	Abductor.Digiti.Minimi, Flexor.Digiti.Minimi.Brevis, Plantar.Interosseus.3
8 **digit 1**	0.25249	Distal.Phalanx.1	Extensor.Hallucis.Longus, Flexor.Hallucis.Longus

**Table 18:** Musculoskeletal modules of hindlimb of normal newborn using AnNA (see Text). ant., anterior; comp., compartment; post., posterior.

ID	p.value	Muscles	Bones
1 **digits 2,3,5**	0	Proximal.Phalanges 2,3, Middle.Phalanges 2,3,5, Distal.Phalanges 2,3,5	**ant. comp.leg:** Extensor.Digitorum.Longus, Fibularis.Tertius, Extensor.Digitorum.Brevis, **deep post. comp. leg**: Flexor.Digitorum.Longus, **foot**: Flexor.Digitorum.Brevis, Quadratus.Plantae, Lumbricals 1,2,4, Plantar.Interosseus 1, Dorsal.Interossei.1,2,3
2 **axial, gluteal**	0.01538	Vertebrae, Ilium, Sacrum	Gluteus.Maximus, Gluteus.Medius, Gluteus.Minimus, Tensor.Fasciae.Latae, Piriformis, Iliopsoas, Sartorius
3 **gluteal, thigh**	0	Pubic, Ischium, Femur, Patella, Tibia	**gluteal**: Obturator.Internus, Obturator.Externus, Gemellus.Superior, Gemellus.Inferior, Quadratus.Femoris, **ant. comp. thigh/quadriceps femoris**: Rectus.Femoris, Vastus.Lateralis, Vastus.Intermedius, Vastus.Medialis, **medial comp. thigh**: Pectineus, Adductor.Brevis, Adductor.Magnus, Adductor.Longus, Gracilis, **hamstrings**: Biceps.Femoris, Semitendinosus, Semimembranosus, Popliteus
4 **tarsals, metatarsals**	0.00028	Navicular, Cuboid, Lateral.Cuneiform, Intermediate.Cuneiform, Medial.Cuneiform, Metatarsals 1,2,3,4	Fibularis.Longus, Tibialis.Anterior, Tiblialis.Posterior, Flexor.Hallucis.Brevis, Adductor.Hallucis
5 **tarsals, digit 1**	0.21666	Fibula, Calcaneus, Talus, Proximal.Phalanx.1,	**superf. post. comp. leg**: Plantaris, Gastrocnemius, Soleus, **lat. comp. leg**: Fibularis.Brevis, **ant. comp. leg**: Extensor.Hallucis.Brevis, **Hallucis**: Abductor.Hallucis
6 **digit 4**	0.04496	Proximal.Phalanx.4, Middle.Phalanx.4, Distal.Phalanx.4,	Lumbrical.3, Plantar.Interosseus 2, Dorsal.Interosseus 4
7 **digit 5**	0.11641	Metatarsal.5, Proximal.Phalanx.5,	Abductor.Digiti.Minimi, Flexor.Digiti.Minimi.Brevis, Plantar.Interosseus 3
8 **digit 1**	0.25249	Distal.Phalanx.1,	Extensor.Hallucis.Longus, Flexor.Hallucis.Longus

**Table 19:** Musculoskeletal modules of left hindlimb of normal 7-month fetus using AnNA (see Text). ant., anterior; comp., compartment; post., posterior.

ID	p.value	Bones	Muscles
1 **digits 2,3,5**	0	Proximal.Phalanges 2,3, Middle.Phalanges 2,3,5, Distal.Phalanges 2,3,5	**Anterior comp. leg**: Extensor.Digitorum.Longus, Fibularis.Tertius, **deep post. comp. leg**: Flexor.Digitorum.Longus, foot: Extensor.Digitorum.Brevis, Flexor.Digitorum.Brevis, Quadratus.Plantae, Lumbricals 1,2,4, Plantar.Interosseus 1, Dorsal.Interossei 1,2,3
2 **tarsals, metatarsals, digit 1**	0	Fibula, Calcaneus, Talus, Navicular, Cuboid, Lateral.Cuneiform, Intermediate.Cuneiform, Medial.Cuneiform, Metatarsals 1,2,3,4, Proximal.Phalanx.1,	**Superf. post. comp. leg**: Plantaris, Gastrocnemius, Soleus, **lat. comp. leg**: Fibularis.Longus, Fibularis.Brevis, **ant. comp. leg**: Tibialis.Anterior, **deep post. comp. leg**: Tiblialis.Posterior, **Hallucis**: Extensor.Hallucis.Brevis, Abductor.Hallucis, Flexor.Hallucis.Brevis, Adductor.Hallucis
3 **gluteal, thigh**	0	Pubis, Ischium, Femur, Patella, Tibia	**Gluteal region**: Obturator.Internus, Obturator.Externus, Gemellus.Superior, Gemellus.Inferior, Quadratus.Femoris, **Ant. comp. thigh/quadriceps femoris**: Rectus.Femoris, Vastus.Lateralis, Vastus.Intermedius, Vastus.Medialis, **medial comp. thigh**: Adductor.Brevis, Adductor.Magnus, Adductor.Longus, Pectineus, Gracilis, **Hamstrings**: Biceps.Femoris, Semitendinosus, Semimembranosus, Popliteus
4 **axial, gluteal**	0.00544	Vertebrae, Ilium, Sacrum	Gluteus.Maximus, Gluteus.Medius, Gluteus.Minimus, Tensor.Fasciae.Latae, Piriformis, Iliopsoas, Sartorius
5 **digit 4**	0.04496	Proximal.Phalanx.4, Middle.Phalanx.4, Distal.Phalanx.4,	Lumbrical.3, Plantar.Interosseus 2, Dorsal.Interosseus 4
6 **digit 5**	0.11641	Metacarpal.5, Proximal.Phalanx.5	Abductor.Digiti.Minimi, Flexor.Digiti.Minimi.Brevis, Plantar.Interosseus 3
7 **digit 1**	0.25249	Distal.Phalanx.1,	Extensor.Hallucis.Longus, Flexor.Hallucis.Longus

**Table 20:** Musculoskeletal modules of right hindlimb of normal 7-month fetus using AnNA (see Text). ant., anterior; comp., compartment; post., posterior.

ID	p.value	Bones	Muscles
1 **axial, gluteal**	0.01538	Vertebrae, Ilium, Sacrum,	Gluteus.Maximus, Gluteus.Medius, Gluteus.Minimus, Tensor.Fasciae.Latae, Piriformis, Iliopsoas, Sartorius
2 **digits 3,5**	0.00798	Proximal.Phalanx.3, Middle.Phalanges 3,5, Distal.Phalanges 3,5	**ant. comp. leg**: Extensor.Digitorum.Longus, Fibularis.Tertius, Extensor.Digitorum.Brevis, **deep post. comp. leg**: Flexor.Digitorum.Longus, foot: Flexor.Digitorum.Brevis, Quadratus.Plantae, Lumbricals 2,4, Plantar.Interosseus 1, Dorsal.Interosseus 3
3 **gluteal, thigh**	0	Pubis, Ischium, Femur, Patella, Tibia,	**gluteal**: Obturator.Internus, Obturator.Externus, Gemellus.Superior, Gemellus.Inferior, Quadratus.Femoris, **ant. comp. thigh, quadriceps femoris**: Rectus.Femoris, Vastus.Lateralis, Vastus.Intermedius, Vastus.Medialis, **medial comp. thigh:** Pectineus, Adductor.Brevis, Adductor.Magnus, Adductor.Longus, Gracilis, **Hamstrings**: Biceps.Femoris, Semitendinosus, Semimembranosus, Popliteus
4 **tarsals, digit 1**	0.16334	Fibula, Calcaneus, Talus, Proximal.Phalanx.1,	**superf. post. comp. leg**: Plantaris, Gastrocnemius, Soleus, **lat. comp. leg**: Fibularis.Brevis, **ant. comp. leg**: Extensor.Hallucis.Brevis, **Hallucis**: Abductor.Hallucis, Flexor.Hallucis.Brevis
5 **tarsals, metatarsals**	0.00064	Navicular, Cuboid, Lateral.Cuneiform, Intermediate.Cuneiform, Medial.Cuneiform, Metatarsals 1,2,3,4	**lat. comp. leg**: Fibularis.Longus, **ant. comp. leg**: Tibialis.Anterior, **deep post. comp. leg**: Tiblialis.Posterior, **Hallucis**: Adductor.Hallucis
6 **digit 4**	0.04496	Proximal.Phalanx.4, Middle.Phalanx.4, Distal.Phalanx.4,	Lumbrical.3, Plantar.Interossei.2, Dorsal.Interossei.4
7 **digit 5**	0.11641	Metatarsal.5, Proximal.Phalanx.5,	Abductor.Digiti.Minimi, Flexor.Digiti.Minimi.Brevis, Plantar.Interosseus 3
8 **digit 2**	0.00773	Proximal.Phalanx.2, Middle.Phalanx.2, Distal.Phalanx.2,	Lumbrical.1, Dorsal.Interosseus 1, Dorsal.Interosseus 2
9 **digit 1**	0.25249	Distal.Phalanx.1,	Extensor.Hallucis.Longus, Flexor.Hallucis.Longus

**Table 21:** Musculoskeletal modules of left hindlimb of T-18 cyclopic fetus using AnNA (see Text). ant., anterior; comp., compartment; post., posterior.

ID	p.value	Bones	Muscles
1 **gluteal, thigh**	0	Pubis, Ischium, Ilium, Femur, Patella, Tibia,	**Gluteal region**: Gluteus.Medius, Gluteus.Minimus, Tensor.Fasciae.Latae, Obturator.Internus, Obturator.Externus, Gemellus.Superior, Gemellus.Inferior, Quadratus.Femoris, **Anterior compartment**: Sartorius, **Quadriceps femoris**: Rectus.Femoris, Vastus.Lateralis, Vastus.Intermedius, Vastus.Medialis, **Medial compartment**: Pectineus, Adductor.Brevis, Adductor.Magnus, Adductor.Longus, Gracilis, **Hamstrings**: Biceps.Femoris, Semitendinosus, Semimembranosus, Popliteus
2 **digits 2,3,4**	0	Proximal.Phalanges 2,3,4, Middle.Phalanges 2,3,4, Distal.Phalanges 2,3,4	Extensor.Digitorum.Longus, Extensor.Digitorum.Brevis, Flexor.Digitorum.Longus, Flexor.Digitorum.Brevis, **deep foot mm**: Lumbricals 1,2,3, Plantar.Interossei 1,2, Dorsal.Interossei 1,2,3,4
3 **tarsals, metatarsals**	0.00028	Navicular, Cuboid, Lateral.Cuneiform, Intermediate.Cuneiform, Medial.Cuneiform, Metatarsals 1,2,3,4	Fibularis.Longus, Tibialis.Anterior, Tiblialis.Posterior, Flexor.Hallucis.Brevis, Adductor.Hallucis
4 **tarsals, digit 1**	0.1636	Fibula, Calcaneus, Talus, Proximal.Phalanx.1,	**Superficial posterior compartment**: Gastrocnemius, Soleus, Plantaris, **Foot**: Quadratus.Plantae, Extensor.Hallucis.Brevis, Abductor.Hallucis,
5 **digit 5**	0.01085	Metatarsal.5, Proximal.Phalanx.5, Middle.Phalanx.5, Distal.Phalanx.5,	Fibularis.Brevis, Fibularis.Tertius, Abductor.Digiti.Minimi, Lumbrical.4, Flexor.Digiti.Minimi.Brevis, Plantar.Interosseus 3
6 **axial**	0.32565	Vertebrae, Sacrum,	Gluteus.Maximus, Piriformis, Iliopsoas
7 **digit 1**	0.25249	Distal.Phalanx.1,	Extensor.Hallucis.Longus, Flexor.Hallucis.Longus

**Table 22:** Musculoskeletal modules of right hindlimb of T-18 cyclopic fetus using AnNA (see Text). ant., anterior; comp., compartment; post., posterior.

ID	p.value	Bones	Muscles
1 **digits 2, 3, 4**	0	Proximal.Phalanx.2,3,4, Middle.Phalanx.2,3,4, Distal.Phalanx.2, 3,4,	Extensor.Digitorum.Longus, Flexor.Digitorum.Longus, Extensor.Digitorum.Brevis, Flexor.Digitorum.Brevis, Lumbricals 1,2,3, Plantar Interossei 1,2, Dorsal.Interossei 1,2,3,4
2 **gluteal/thigh**	0	Pubis, Ischium, Ilium, Femur, Patella, Tibia,	**Gluteal**: Gluteus.Medius, Gluteus.Minimus, Tensor.Fasciae.Latae, Obturator.Internus, Obturator.Externus, Gemellus.Superior, Gemellus.Inferior, Quadratus.Femoris, **anterior comp. thigh**:. Sartorius, **quadriceps femoris**: Rectus.Femoris, Vastus.Lateralis, Vastus.Intermedius, Vastus.Medialis, **medial comp. thigh**: Pectineus, Adductor.Brevis, Adductor.Magnus, Adductor.Longus, Gracilis, **Hamstrings**: Biceps.Femoris, Semitendinosus, Semimembranosus
3 **digit 5**	0.00893	Metatarsal.5, Proximal.Phalanx.5, Middle.Phalanx.5, Distal.Phalanx.5,	Abductor.Digiti.Minimi, Lumbrical.4, Flexor.Digiti.Minimi.Brevis, Plantar.Interossei.3
4 **tarsals/digit 1**	0.16787	Fibula, Calcaneus, Talus, Proximal.Phalanx.1	**Lat. comp. leg**: Fibularis.Brevis, **Ant. comp. leg**: Fibularis.Tertius, **Superf. post. comp. leg**: Gastrocnemius, Soleus, Plantaris, **Foot**: Quadratus.Plantae, Extensor.Hallucis.Brevis, Abductor.Hallucis
5 **tarsals, metatarsals**	0.00028	Navicular, Cuboid, Lateral.Cuneiform, Intermediate.Cuneiform, Medial.Cuneiform, Metatarsals 1, 2, 3, 4	Fibularis.Longus, Tibialis.Anterior, Tiblialis.Posterior, Flexor.Hallucis.Brevis, Adductor.Hallucis
6 **axial**	0.32565	Vertebrae, Sacrum,	Gluteus.Maximus, Piriformis, Iliopsoas
7 **digit 1**	0.25249	Distal.Phalanx.1,	Extensor.Hallucis.Longus, Flexor.Hallucis.Longus

**Table 23:** Musculoskeletal modules of left hindlimb of ancencephalic fetus 1 using AnNA (see Text). ant., anterior; comp., compartment; post., posterior.

ID	p.value	Bones	Muscles
1 **digit 1, tarsals**	0	Fibula, Talus, Calcaneus, Navicular, Cuboid, Medial.Cuniform, Intermediate.Cuniform, Lateral.Cuniform, Metatarsal.1, Proximal.Phalanx.1, Distal.Phalanx.1,	**Anterior comp. leg**: Tibialis.Anterior, Extensor.Hallucis.Longus, **lat. comp. leg**: Fibularis.Longus, Fibularis.Brevis, **superf. post. comp. leg**: Gastrocnemius, Soleus, Plantaris, **deep post. comp. leg**: Tibialis.Posterior, Flexor.Hallucis.Longus, **Hallucis**: Abductor.Hallucis., Adductor.Hallucis, Muscle.of.Henle, Flexor.Hallucis.Brevis, Extensor.Hallucis.Brevis
2 **axial, gluteal, thigh**	0	Vertebrae, Sacrum, Pubis, Ischium, Ilium, Femur, Patella, Tibia	**Gluteal region**: Gluteus.Maximus, Gluteus.Medius, Gluteus.Minimus, Tensor.Fasciae.Latae, Piriformis, Superior.Gemellus, Obturator.Internus, Inferior.Gemellus, Obturator.Externus, Quadratus.Femoris, **Anterior compartment**: Sartorius, Iliopsoas, **Quadriceps femoris**: Rectus.Femoris, Vastus.Lateralis, Vastus.Medialis, Vastus.Intermedius, **Adductor/medial compartment**: Pectineus, Adductor.Longus, Adductor.Brevis, Adductor.Magnus, Gracilis, **Hamstrings**: Biceps.Femoris., Semitendinosus, Semimembranosus, Popliteus
3 **digit 4 & 5**	2.00E-05	Metatarsals 4,5, Proximal.Phalanges 4,5, Middle.Phalanges 4,5, Distal.Phalanges 4,5	**Anterior compartment leg**: Extensor.Digitorum.Longus, Fibularis.Tertius, **Posterior compartment leg**: Flexor.Digitorum.Brevis, **Plantar side**: Abductor.Digiti.Minimi, Lumbricals.3,4, Flexor.Digiti.Minimi.Brevis, Dorsal.Interossei 3,4, Plantar.Interossei 2,3
4 **digit 2 & 3**	4.00E-05	Metatarsals 2,3, Proximal.Phalanges 2,3, Middle.Phalanges 2,3, Distal.Phalanges 2,3,	**Posterior compartment leg**: Flexor.Digitorum.Longus, **Plantar side**: Lumbricals 1,2, Quadratus.Plantae, Dorsal.Interosseous.1, Dorsal.Interosseous.2, Planter.Interosseous.1, Extensor.Digitorum.Brevis

**Table 24:** Musculoskeletal modules of right hindlimb of ancencephalic fetus 1 using AnNA (see Text). ant., anterior; comp., compartment; post., posterior.

ID	p.value	Bones	Muscles
1 **patella & quadriceps femoris**	0.00279	Patella	Rectus.Femoris, Vastus.Lateralis, Vastus.Medialis, Vastus.Intermedius
2 **thigh**	0.01995	Ischium, Fibula, Tibia, Talus, Calcaneus	Quadratus.Femoris, Sartorius, Gracilis, Biceps.Femoris, Semitendinosus, Semimembranosus, Popliteus, Gastrocnemius, Soleus, Plantaris, Tibialis.Posterior
3 **gluteal thigh**	2e-04	Pubis, Ilium, Femur,	Gluteus.Maximus, Gluteus.Medius, Gluteus.Minimus, Tensor.Fasciae.Latae, Superior.Gemellus, Obturator.Internus, Inferior.Gemellus, Obturator.Externus, Pectineus, Adductor.Longus, Adductor.Brevis, Adductor.Magnus
4 **digit 5**	0.09689	Metatarsal.5, Proximal.Phalanx.5, Middle.Phalanx.5, Distal.Phalanx.5,	Fibularis.Tertius, Fibularis.Brevis, Abductor.Digiti.Minimi, Lumbrical.4, Flexor.Digiti.Minimi.Brevis
5 **axial**	0.40683	Vertebrae, Sacrum	Piriformis
6 **tarsals & digit 1**	0.00174	Navicular, Cuboid, Medial.Cuniform, Intermediate.Cuniform, Lateral.Cuniform, Metatarsal.1, Proximal.Phalanx.1	Tibialis.Anterior, Fibularis.Longus, Abductor.Hallucis, Adductor.Hallucis.Oblique.Head, Muscle.Of.Henle, Extensor.Hallucis.Brevis
7 **digit 1**	0.3042	Distal phalanx 1	Extensor.Hallucis.Longus, Flexor.Hallucis.Longus, Quadratus.Plantae,
8 **digits 2, 3, 4**	2e-05	Metatarsal 3, Proximal.Phalanx 3, Middle.Phalanges 2,3,4, Distal.Phalanges 2,3,4	Extensor.Digitorum.Longus, Flexor.Digitorum.Longus, Extensor.Digitorum.Brevis, Flexor.Digitorum.Brevis, Lumbricals 2,3, Plantar.Interosseous 2
9 **digit 2**	0.16449	Metatarsal 2, Proximal Phalanx 2	Dorsal interossei 1,2, Plantar interosseous 1
10 **digit 4**	0.2146	Metatarsal 4, Proximal Phalanx 4	Dorsal interosseous 4, Plantar interosseous 3

### Musculoskeletal parameters and modularity of the normal and abnormal heads, FLs and HLs

Concerning Table 1, the parameters measured were: the total number of nodes (*N*) and links (*K*), density of connections (*D*), mean clustering coefficient (*C*), mean shortest path length (*L*), heterogeneity of connections (*H*) (see Methods for more details). *Network nodes (N)* are interacting components of the anatomical structure, for example, bones and muscles. *Network links (K)* are interactions or relations among components, for example, physical contacts. Links may directly or indirectly contribute to biological processes, such as growth or function. *Density of connections (D)* concerns the richness or complexity of the anatomical structure as well as anatomical integration, as it relates to the number of interactions. *Mean clustering coefficient (C)* concerns anatomical integration, as it relates to functional and/or developmental interdependence among triplets of components. Mean shortest path length (*L*) also concerns anatomical integration, as it relates to the effective proximity between components that allows coordination, independently of their spatial or geometric distance. Lastly, heterogeneity of connections (*H*) is related to differentiation or anisomerism of components in the anatomical structure, as well as to irregularity, as it contrasts with regular structures (zero heterogeneity). Regarding Table 2, within *modularity*, Q measures the quality of the modularity results as a whole: Q < 0.3 is low modularity; Q > 0.3 starts to be high modularity, the higher the Q the more modular; Qerror is a dispersion of Q, basically to assess if it is <or> than 0.3; so, if Q - Qerror > 0.3 then one is more confident that the partition of the network in these modules (all together) is better than expected at random.

In terms of the number of musculoskeletal structures of the individuals compared in the present work (Table 1: N), it can be said that despite some exceptions, in general the number of structures is lower in the fetuses with congenital malformations, when compared to the condition seen in the normal 7-month-old fetus. For instance, within the 11 cases compared, this happens in eight of them. So, while the normal 7-month- old fetal head has 120 structures, the T18 head has 94, the anencephalic fetus 2 head has 72, and the anencephalic fetus head 1 has 58 (Table 1; see also SI). Regarding the FL, the two T18 FLs and the right anencephalic 1 FL have less structures than the FLs of the normal 7-month- old fetus, but the left FLs of the anencephalic fetus 1 has the same number. Concerning the HLs only the left HL of the anencephalic fetus 1 has less structures than the normal 7-month- old fetus (Table 1; see also SI). A similar pattern is seen concerning the number of connections between these structures (Table 1: K): cases of malformations almost always have a lower K. However, it should be noted that apart from clear left-right asymmetries concerning the values of N and K of the FLs and/or HLs of individuals with malformations (see Table 1 and below), there are also marked differences in these values between individuals displaying the same type of malformation: e.g., the head of the anencephalic fetuses 1 and 2 have a K of 102 vs 172 (Table 1).

Interestingly, although the generally lower number of structures and connections in the abnormal fetuses does not related to a clear increase or decrease of the number of modules (of the 11 pairs of comparisons referred to above, in 6 the number of modules is lower and in 5 is higher), one does see a clear, but reverse, pattern regarding network complexity, as measured by D (Table 1). That is, the network complexity is always higher in the pathological cases, for all the head and FLs, with exception to the left FL of the anencephalic fetus 1. Interestingly, concerning the HLs, all of them, both in the normal and abnormal individuals, have in general a similar D (Table 1), which goes in line with the idea that in general HLs have both less variations in the normal population and less defects in cases of malformation: these issues will be discussed in the Discussion, below.

### The musculoskeletal network modules of the normal and abnormal heads

In this paper we provide an updated, more polished version, of the anatomical networks of the normal human adult head (Fig. 1; Table 3), in contrast to that we published in a previous study^11^. For instance, the arytenoideus transversus was coded as a single muscle, because there are no separate left and right transverse arytenoid muscles; accordingly, the arytenoideus obliquus muscle was also coded as a single muscle in the matrixes done for this study (see SI). By doing this, the musculoskeletal modules obtained are functionally and/or developmentally more cohesive (Table 3; Fig. 1). For instance, instead of a “lower jaw/inner ear” musculoskeletal module, there is now an “eye/mastication” module (Table 3; Fig. 1), thus mirroring the results of recent studies supporting the idea that the extraocular muscles are developmentally and evolutionarily related to the muscles of mastication^17^.

There are some general similarities between the normal newborn (Fig. 2; Table 4) and normal adult head musculoskeletal networks (Fig. 1; Table 3), but there are also some specific differences. For instance, the “occipital” module includes the left temporal bone and the left auricularis posterior and stylopharyngeus muscles, while it includes the right ones in the normal adult head. Additionally, in the normal newborn the trapezius is also included in this “occipitalis” module, and not in a separate module as in the normal adult head. The right auricularis posterior and stylopharyngeus are included in a right module that includes several muscles of facial expression, muscles of mastication, and tongue muscles in the normal newborn, while in the adult head the tongue muscles are included in a module together with the hyoid muscles. In general, the musculoskeletal modules of the normal 7-month-old fetus head are similar to those of the normal newborn head (Table 5).

In contrast to the normal newborn, the skeletal system of the T18 cyclopic fetus head has only 13 bones/cartilages (about half the normal number); it also has fewer muscles (96 vs. 110) than the normal newborn head, as well than the normal 7-month-old fetus head (see SI). This indicates that some structures, as well as bone-muscle attachments, of the head are lost in this pathological condition. As a result, there is a major difference between the modular organization of the normal head and that of the cyclopic head (Table 6; Fig. 3), which occurs in the skeletal system and at the interface of that system and the muscular system. In fact, the T18 head as only four major musculoskeletal modules: an “eye-mastication” one including the fused central bone, the mandible, the sphenoid, and various eye and mastication muscles; a “skull-mastication-facial” module including various skull bones plus some facial and mastication muscles; and left and right “facial” modules including only facial muscles (Table 6; Fig. 3).

The musculoskeletal network organization of the head of the anencephalic fetus 1 (Fig. 4; Table 7) and 2 (Table 8) are very different from those of the normal newborn and T18 cyclopic heads. The head of the anencephalic fetus 1 has five major modules: “left upper-mid face”, “left skull-mandible-lower face”, “right mandible-lower face”, “right upper-mid face”, and “skull-digastric-neck muscles” (Fig. 4; Table 7). The head of the anencephalic fetus 2 has eight major modules: “hyoid and tongue”, “face and neurocranium”, “right face and facial muscles”, “left face and facial muscles”, “right cranium and trapezius”, “left temporal and neck muscles”, “right temporal and neck muscles”, and “left cranium and trapezius” (Table 8).

### The musculoskeletal network modules of the normal and abnormal FLs

The normal newborn modules are almost exactly the same ones as in the normal adult (Tables 9 and 10; Fig. 5), the only difference being that the deltoideus is part of the “shoulder girdle” module in the adult because it is often blended with the pectoralis major, while in the newborn the deltoideus is part of the “arm/forearm” module. The modules of the left and right side of the normal 7-month-old fetus (Tables 11 and 12) are essentially the same, and they are also basically the same to those of the normal newborn and adult, the main difference being that the “digit 5” and “carpals/digit 5” modules of the normal newborn are grouped into a single “carpals/digit 5” module in the 7-month-old fetus. Therefore, the forelimb of the fetus has eight modules and not nine, as does the FL of the normal newborn and adult. It is very interesting to see that the flexor pollicis longus and extensor pollicis longus consistently form a module together with the distal phalanx of thumb in the normal fetal, newborn, and adult FLs, because these two muscles are said to have played a major role, very likely coupled together, in human evolution (see, e.g., Diogo *et al*.^19^).

The modules of the left T18 FL (Fig. 6; Table 13) are considerably different from those of the FLs of the 7-month-old fetus. Firstly, there are “shoulder girdle A” and “shoulder girdle B” modules in the left T18 FL while the normal 7-month-old fetus has a single “shoulder girdle” module. The extra “shoulder girdle B” module of the left T18 FL has some structures that are included in the single “shoulder girdle” module of the normal 7-month-old fetus (e.g., scapula) and others that are part of the “arm/forearm” module of the latter fetus, such as muscles attached to the scapula (e.g., infraspinatus, supraspinatus). Interestingly, the biceps brachii, which is part of the “arm/forearm” of the normal 7-month-old fetus, is integrated into the “shoulder A” module of the left T18 forelimb because of its abnormal association with the pectoralis major (see SI; see also Smith *et al*.^17^, for more details). Lastly, the “distal phalanx 1” module - which includes the flexor and extensor pollicis longus and the distal phalanx 1 and is consistently found in the normal fetal, newborn, and adult phenotype, as noted above - is completely integrated with the structures that are part of the “arm/forearm” module of the normal fetus, to form a peculiar “arm/forearm/digit 1” module in the left T18 FL. That is, the number of modules in the left T18 FL and in the FLs of the normal 7-month fetus is the same (eight), but half of these modules are significantly different within the two fetuses (compare Tables 11–13).

The right T18 FL modules (Fig. 7; Table 14) are even more different from those of the normal 7-month-old fetus FLs than those of the left T18 FL. For instance while in the normal fetus there is a “shoulder girdle” module, in the right T18 FL there is a “shoulder girdle” module plus a peculiar “posterior shoulder” module; while in the former there is a “digits 2 and 3” module, in the latter there are separate “digit 2” and “digit 3” modules; while in the former there is a “carpals/digit 5” module, in the latter there are separate “carpals/digit 5” and “digit 5” modules. As a result, there is also a marked left-right asymmetry concerning the network modules of the FLs in the T18 fetus.

Of the two FLs of the anencephalic fetus 1 (Figs 8 and 9; Tables 15 and 16), the right is clearly the more abnormal in terms of its gross anatomy, lacking a radius and a digit 2^18^. Accordingly, in terms of its modules, the left FL displays a mix of modules that are often seen in either the prenatal or adult normal phenotype (e.g., “carpals/digit 5” and “digit 2”) and that are quite unique. Examples of quite unique modules is the presence of a “pollicis” module including only the abductor pollicis longus and brevis and the extensor pollicis brevis, of a module that includes both the “carpals + digit 1” module plus the “digit 1” modules often seen in the normal phenotype, and a module that includes both the “shoulder girdle” module plus the “shoulder girdle/arm/forearm” module often seen in the normal phenotype. Interestingly, also on the left FL of the anencephalic fetus 1 there is a very peculiar module that includes only the occipital bone, vertebrae, and the rhomboid complex, which is the result of the presence of an extra, abnormal muscle in this complex connecting the vertebrae to the head: the rhomboideus occipitalis (see SI and Alghmadi *et al*.^18^). This latter muscle is often seen in humans with severe birth defects (see, e.g., reviews by Smith *et al*.^17^; Diogo *et al*.^20^) and is commonly present in non-human primates^21–25^.

The right FL of the anencephalic fetus 1 is, as expected due to its marked gross anatomical malformations, the most peculiar one in terms of number of modules: while all FLs of the normal individuals, as well as of the T18 fetus, have 8 or 9 modules, and the left FL of the anencephalic fetus 1 has 7, the right FL of this latter fetus has only 5. However, despite its very modified gross anatomy, within its 5 modules, two are similar to those present in the left FL of the same fetus (“carpals/digit 5” and “shoulder girdle/arm/forearm”), and one - not seen in left FL of the same fetus - is seen in at least some FLs of normal individuals (“digit 4”). That is, only the two other modules of the right FL of the anencephalic fetus 1 are truly peculiar: one (“digit 1”) includes only bones, i.e., the metacarpal 1 and proximal and distal phalanges of the thumb, and the other (“carpals/digit 3”) includes a combination of structures not seen in any other FL.

### The musculoskeletal network modules of the normal and abnormal HLs

The only difference between the newborn HL vs. adult HL modules (Tables 17 and 18; Fig. 10) is that in the newborn the ilium, ischium, and pubis are separate bones, so the ischium and pubis remain part of the “gluteal/thigh” module that includes the whole hip bone in the adult, while the ilium is included, together with the gluteus minimus, gluteus medius, and tensor fasciae latae that attach onto it, into the axial module, thus forming an “axial/gluteal” module. The network modules of the right HL of the normal 7-month-old fetus (Table 20) are almost exactly the same as those of the normal newborn, as expected because the ischium, ilium, and pubis are also separated bones, the single difference being that in the normal newborn (and adult) there is a “digits 2, 3 and 5” module, while in the right HL of the normal fetus there is a “digits 3 and 5” module plus a separate “digit 2” module, i.e., there are nine modules in total instead of eight. Remarkably, in the left HL of the normal fetus (Table 19) there are only seven modules, the only difference with the normal newborn (and adult) HLs being that in the latter there is a “tarsals/metatarsals” module plus a “tarsals/digit 1” module, while in the left HL of the normal fetus these two modules are included in a single “tarsals/metatarsals/digit 1” module. But in overall the modular organization of the HLs of the normal 7-month-old fetus, newborn, and adult individuals are essentially very similar, as expected, i.e., one bigger module can be eventually split into two, but there are no truly completely different, peculiar modules, in the normal fetus.

What is perhaps more surprising is that the modules of both the left and right HLs of the T18 cyclopic fetus (Tables 21 and 22; Fig. 11) are also very similar to the ones of the normal adult and newborn HL, the main difference being that in the normal adult there is a “digits 2, 3, and 5” module, a separate “digit 4” module, and a separate “digit 5 module”, while in the T18 there is a “digits 2, 3, and 4” module and then a single module for digit 5. That is, the normal adult HL has one more module in total than the left and right T18 HLs (8 vs 7), but the overall network modules of all these HLs are in general very similar to each other. In fact, the network modules of the left and right HLs of T18 are almost the same, with the interesting difference that in the left HL the fibularis tertius and brevis are part of the “5th digit” module, as would be somewhat logically to expect, while in the right HL they are part of the “tarsal/1st digit” module.

In contrast, the modules of both the left and right HLs of the anencephalic fetus 1 (Tables 23 and 24; Figs 12 and 13) are very different from each other, and also from the HLs of any other human individual studied by us. On the left HL of this fetus there are only four modules: one (“axial/gluteal/thigh”) mainly corresponds to the axial module plus the gluteal/thigh module often seen in the normal phenotype; one (“tarsals/digit 1”) mainly corresponds to the tarsals/digit 1 module plus the digit 1 module also often seen in the normal phenotype; and the two other modules (“digits 2 and 3” and “digits 4 and 5”) are unique within any human HL studied by us. Regarding the right HL of this anencephalic fetus 1, it has a higher number of modules than any other human HL studied by us: ten. Seven of these ten modules are somewhat similar to seven of the nine modules of the right HL of the normal 7-month-old fetus, but the other three are quite unique: “patella/quadriceps femoris” module including only these structures, separate “thigh” module, and “digits 2, 3, and 4” module.

## Discussion

As noted in our previous publications on the gross anatomy of the T18 fetus^17,26–28^ and the anencephalic fetuses^18^ included in the AnNA of the present work, the more marked differences between these fetuses and the ‘normal’ (see below) phenotype of a 7-month-old fetus are found in the head (Figs 1–4). This was to be expected as the abnormal fetuses have severe head malformations: anencephaly (Fig. 4) and cyclopia (Fig. 3). Within limbs, the one that is clearly more abnormal, in terms of gross anatomy, is the right FL of the anencephalic fetus 1, in which both the radius and digit 2 are missing, with many forearm/hand muscles missing and others that normally attach to digit 2 inserting instead onto the adjacent digits (Fig. 9; for more details see SI and Alghamdi *et al*.^18^). As also discussed in those previous studies, and in Diogo *et al*.^20^, despite those malformations, the *gross anatomical analyses* of these and other abnormal human fetuses actually reinforce Alberch’s ill-named “logic of monsters”, in the sense that even individuals with severe and different types of syndromes/conditions (e.g., cyclopic vs. anencephalic) display at least some very similar gross anatomical patterns/configurations. For instance, the FLs almost always display a higher number of defects than the HLs, mirroring what happens in terms of the variations in the ‘normal’ population (see Smith *et al*.^17^ and Diogo *et al*.^20^ for detailed discussions on this subject).

Updated discussion of the “logic of monsters” hypothesis proposed by Alberch was given in Diogo *et al*.^20,28^. In short, this hypothesis argues that there is a general parallelism between the variation/defects in normal/abnormal individuals of a species and the fixed diversity observed in wild type phenotypes of related species. This parallelism is achieved through regulation of a conserved developmental program (i.e., the set of genetic and epigenetic interactions), such that the structure of these internal interactions constrains the realm of possible variation upon which selection can operate. This theory contrasts with the “lack of homeostasis” proposed by Shapiro^5^, which was mainly formulated based on studies of human trisomy, and that argues that in such individuals the presence of a whole extra functioning chromosome, or a large chromosome segment, causes a generalized disruption of the evolved genetic balance. Because of the obligatory integration of the entire genotype, this disruption affects the products of both the trisomic chromosome and of other chromosomes. This results in decreased developmental and physiological buffering against genetic and environmental forces, leading to a generalized decreased developmental and physiological homeostasis. This hypothesis therefore suggests that defects are in general more *random* and disorganized due to a general lack of homeostasis (e.g., very often leading to left-right asymmetry), while the “logic of monsters” predicts that defects are more “logical” and “constrained” because constraints are in general still kept by internal homeostasis even in cases of abnormal development^20^. However, although the two hypotheses are substantially different in theory, i.e., Alberch’s “order/logic” vs. Shapiro’s “randomness/chaos” in a very simplified and dualistic way, in practice they have nuances that are actually compatible in some cases, as is actually very much the case with the results of our AnNA of the present work, as will be discussed below.

So, how do our AnNA quantitative results compare with the qualitative gross anatomical comparisons that we have previously published about the same human normal and abnormal individuals included in the present study? The T18 fetus and anencephalic fetal heads and limbs have fewer skeletal and muscular elements than the normal newborn and 7-month-old fetus heads, very likely due to the marked disruption of normal development leading to various absent or fused skeletal and muscular elements. Interestingly, one can also see a clear, but reverse, pattern regarding network complexity, as measured by D: the network complexity is always higher in the pathological cases, for all heads and FLs, with exception of the left FL of the anencephalic fetus 1 (Table 1). Also, the different pattern seen concerning the HLs - all of them, both in the normal and abnormal individuals, have in general a similar D (Table 1) - supports the idea that usually HLs have both less variations in the normal population and less defects in cases of malformation, as will be further discussed below. This is a typical example that is often provided to support Alberch’s “logic of monsters” because it concerns similarities about normal and abnormal development. Moreover, a similar link between number of structures and network complexity has also been shown to occur in the evolution of tetrapods (e.g., concerning the skull) in general: a decrease of the number of bones does not result in a decrease of network complexity, as could be expected, but instead to a decrease of network complexity (recently reviewed by Esteve-Altava^12,13^). Therefore, this might be a further case supporting Alberch’s “logic of monsters” hypothesis, in the sense that it is a parallel trend occurring both within the macroevolution of normal phenotypes and within the occurrence of congenital malformations.

On the other hand, our AnNA results do seem to indicate that network organization might be more sensitive to smaller changes than is the simple (gross anatomical) presence/absence of muscles, for instance. For example, we do see a more different/chaotic organization within the left-right asymmetries - which are an important component in the Shapiro’s “lack of homeostasis” hypothesis - of the limbs of the fetuses included in the present work than what would be expected from the superficial, gross anatomical observations of these limbs. For instance, the number and overall configuration of the bones, cartilages, and muscles, and their attachments, of the left vs. right normal 7-month-old fetus HLs dissected by us are in general similar, with some exceptions (see SI). However, those few exceptions result in a significantly different modular organization in these HLs, e.g., the left HL of this 7-month-old fetus has seven modules while the right HL has nine (Table 2). Still, as noted above, even at a network level these differences are still very minimal: the two extra modules of the right side are simply the result of a split of modules of the left side into two. That is, in this case there are no truly different modules as they do not include structures from three or more different modules found in other limbs.

One question that might arise is: is this 7-month-old fetus really ‘normal’, if there are even some gross anatomical differences between the musculoskeletal system of its right vs. left limbs? One could argue that because the fetus died during the 7th month of pregnancy, it might not be ‘normal’. This issue is related to a much-neglected subject in comparative anatomy: the occurrence of anatomical variations within the normal population. As explained in detail in previous works^20^, there are in fact almost always some musculoskeletal - particular muscular - variations in ‘normal’ human bodies, including notable left-right differences, which unfortunately are too often ignored in the literature. In this sense, the left-right differences seen in this 7-month-old fetus are not at all out of the norm for the normal population. This is precisely why we decided to code the limbs of both sides of this fetus, as well as of the anencephalic and cyclopic fetuses, in the matrices of SI1: to also allow a more complete and direct comparison between the left and right sides of all these fetuses, as explained in the section Methods. In fact, there is a marked left-right asymmetry concerning the network modules of the FLs of the T18 fetus (see above), which, again, is more clearly noted in the network modules than it was detected using simply gross anatomical comparisons^17–20^. There is an even higher level of left-right asymmetry concerning the network modules of the HLs of the anencephalic fetus 1, with the left one having four modules and the right one having ten modules. This asymmetry and apparent arbitrariness could be explained by the “lack of homeostasis” hypothesis proposed by Shapiro^5^.

When we put these results together, they do seem to make sense in light of what we now know regarding the more holistic views of systems biology, network theory, and chaos theory, in which a small change can result in a big alteration (e.g., a “butterfly flying can lead to the collapse of a bridge”). So, even the absence of a muscle, or the presence of a new muscle, i.e., *a single gross anatomical change*, can in theory lead to a very different type of network organization of the whole system, or in this case of the left vs. right sides of that system. A further example is that the network modules of the left and right HLs of the T18 fetus are very similar to each other, but there is an interesting difference: in the left HL the fibularis tertius and brevis are part of the “5th digit” module, as would be somewhat logically to be expected, while in the right HL they are part of the “tarsal/1st digit” module. This might seem a paradox at first side, but actually does exemplify the point of systems biology and network theory, and chaos theory more specifically, according to which a small change can result in a relevant difference. In this case that in the right HL of the T18 fetus the fibularis tertius attaches directly to the ulna, contrary to the left HL of this fetus, ‘attracts’ this muscle - and thus also the fibularis brevis, also connected to the fibula - to the module including the fibula (“tarsal/1st digit”). Therefore, on one hand, contrary to the gross anatomical shape and configuration, one does see more chaotic patterns within the network organization of individuals with malformations. On the other hand, in general this ‘chaos’ still occurs with a certain overall ‘logic’, i.e., the fibularis brevis and longus do not form a module with thigh or gluteal muscles, for instance, as it could occur if the system was really lacking any kind of order/homeostasis. This idea is further reinforced when one observes the modules of both the left and right HLs of the anencephalic fetus 1, which display significant differences when compared to the normal phenotype (see above), but still retain some of the modules that are also present in normal individuals or that are simply the result of an addition of two modules seen in the normal phenotype.

In fact, it is important to note that the “logic of monsters” hypothesis^1^ and the “lack of homeostasis” hypothesis^5^ are in marked opposition to each other, but that, as previously noted, they are not mutually exclusive^20,28^. For example, the “logic of monsters” explains why some abnormal fusion of muscles (i.e. between muscles from different anlagen: e.g., flexor pollicis brevis and opponens pollicis; extensor pollicis brevis and abductor pollicis longus) is also present as normal variants in human populations and as the wild-type phenotype in various non-human primates^25^. In contrast, the “lack of homeostasis” explains why these muscles fuse to other muscles derived from a different anlage (e.g., abductor pollicis longus) and form muscular modules that include muscles derived from three distinct anlagen and are innervated by two different nerves^25^. Actually, it is true that in general the left-right asymmetries seen in the abnormal fetuses, discussed above, are probably due to a disruption of the general developmental mechanisms involved in the integration between the right and left limbs, as predicted by the “lack of homeostasis” theory. However, if there was a general developmental disruption throughout the whole body, it would be very difficult to explain why even defective muscles that form the abnormal muscle modules in the left and right HLs of the anencephalic fetus 1 and T18 fetus, and the left and right FLs of the T18 fetus and left FL of the anencephalic fetus 1, share gross anatomical configurations that are in general similar to those seen in the normal phenotypes.

Another example of how both the “logic of monsters” and “lack of homeostasis” can be combined is the presence of a supernumerary muscle in the left and right T18 FLs, the musculus interosseus accessorius (Tables 13 and 14, Figs 6 and 7; SI). The presence of such a peculiar, abnormal extra muscle, described in detail by Smith *et al*.^17^, is an example of a more unexpected, “lack of homeostasis” type of defect, but its bilateral presence is a hallmark of the “logic of monsters” theory. This combination between ‘order’ and ‘chaos’ is also clearly seen in the right FL of the anencephalic fetus 1, which is highly modified, missing a radius and digit 2 (see above). As expected, this is the FL with the most peculiar modules in terms of number of modules, but at the same time two of its five modules are similar to those seen in the left FL of the same fetus, and one is not seen in that left FL but is seen in at least some FLs of normal individuals (“digit 4”).

In summary, our previous works have shown that superficial gross anatomical analyses of these specimens strongly support the “logic of monsters” hypothesis, in the sense that there is an ‘order’ or ‘logic’ within the gross anatomical patterns observed in both the normal and abnormal individuals. Interestingly, the results of the AnNA done in the present study reveal a somewhat different pattern: at least concerning the musculoskeletal modules obtained in our AnNA, we observe a combination between the “logic of monsters” and the “lack of homeostasis” hypotheses. For instance, as predicted by the latter hypothesis, we found a high level of left-right asymmetry in the FLs and/or HLs of the abnormal cyclopic trisomy 18 and anencephalic human fetuses. That is, a network analysis of the organization of/connection between the musculoskeletal structures of these fetuses reveals a more “chaotic” pattern than that detected by superficial gross anatomical comparisons.

## Methods

### Brief introduction to anatomical network analysis (AnNA)

As described in more detail in our previous, recent works^6–15^, AnNA is the study of the connectivity patterns that define the morphological organization of anatomies using tools and statistics borrowed from network theory. The quantitative results of AnNA directly address issues pertaining to modularity, integration, complexity, and evolvability. The first step of AnNA is to code the absence (“0”) or presence (“1”) of contact among anatomical elements (bones and/or muscles) using adjacency (i.e., neighbor: neighbor) Excel matrices, as the ones included in the SI (see also subsection below). Then, with the data compiled in the matrices one often builds three types of network models: (a) skeletal, with bones and their contacts; (b) muscular, with muscles and their contacts; and (c) musculoskeletal, with bones, muscles and their contacts. As explained in the present paper, for this work we focus on the third type of model, the musculoskeletal one. Within these obtained models, in AnNA we typically then perform modularity analyses and phylogenetic analyses using the R packages and identify connectivity modules. A connectivity module is a group of anatomical elements with more connections among them than to any other elements^6–15^. To assess modularity in AnNA, we first often evaluate the likelihood of modules forming by quantifying their connectivity distribution (P*k*), clustering coefficient distribution (C*k*), and small-world organization. A prerequisite to identify clear, biologically meaningful modules in AnNA is the presence of right-skewed P*k* and C*k* and a small-world organization, because these indicate a non-random connectivity pattern that promotes the emergence of anatomical modules. To validate our results, we then typically perform the P*k* and C*k* goodness of fit tests on four different theoretical distributions: Poisson, uniform, exponential, and power-law^6–15^; the organization in bone, muscle and musculoskeletal networks is often analyzed by comparing their clustering coefficient (C) and path length (L), as will be explained in more detail below. Regarding the assessment of integration within modules, a system with modules is free to change in many directions, so (structural) intra-module integration is measured using the network modularity Q-value. For each potential partition, we typically quantify Q: a quality index that quantifies how well a potential partition groups the network nodes compared to other possible partitions^6–15^. If the number of connections among nodes in the same module is not higher than expected at random then Q = 0, otherwise Q > 0: the higher the Q, the better the partition. More specific details, about the particular methods used for the present work, are given in the subsections below.

#### Anatomical matrices

The gross anatomical data used to code the matrices included in the SI of the present work were gathered from our previous publications on the gross anatomy of the normal adult and newborn human phenotype^19–25,29–31^, the T18 fetus (7 months of gestation; male)^17,26,27^ and the normal fetus (7 months of gestation; female) plus the anencephalic fetus 1 (7 months of gestation; male) an fetus 2 (almost 9 months of gestation; female)^18,32^. No new dissections were done for the present study. The way in which we convert gross anatomical data to the anatomical matrices provided in the SI, and then undertake the steps mentioned in the paragraphs below, was explained in detail by Esteve-Altava and colleagues^6–15^. As discussed in more detail in the section Discussion, the coding of the FL and HL of both the normal adult and normal newborn in the matrices of SI was based on the most common phenotype know for these stages, following anatomical atlases (for a recent review, see Diogo *et al*.^30^). In contrast, because the muscles of phenotypically normal 7-month-old fetuses have been much less studied, we decided to code both the left and right FLs and HLs of a normal 7-month-old fetus previously dissected by us^18,32^, as we did for the anencephalic and cylopic fetuses (see SI and Tables 11–16 and 19–24). Apart from taking into account the often neglected fact that phenotypically humans usually do have left-right variations^20^, the decision of including both the left and right sides of this 7-month fetus allows, moreover, a more complete and direct comparison between the left and right sides of all the fetuses included in the present study (normal, anencephalic and cyclopic: see Discussion for more details).

#### Network modeling

For the present work, we built unweighted, undirected network models of the anatomical systems described above. For the skeleton, the nodes of the network and the links connecting them formalized the bones and their pairwise articulations in the anatomy. For the musculature, the nodes and links formalized the muscles and their blends and other muscle-onto-muscle insertions. Musculoskeletal networks combined both the skeletal and the muscular network into a single network model, in which bones and muscles were likewise formalized as nodes and all types of physical contacts were formalized as links. This level of abstraction allows comparing the topological organization of these anatomical systems.

#### Measuring network parameters

To characterize quantitatively the topology of the anatomical systems studied in the present work through their network models we measured a set of six network parameters: number of nodes, number of links, density of connections, mean clustering coefficient, mean path length, and heterogeneity. Such parameters are well-described in the network science literature. For further mathematical details and how they are interpreted in the context of anatomical studies see Rasskin-Gutman and Esteve-Altava^8^). The number of nodes and links measure (as counts) the actual number of anatomical parts and physical connections among them. Number of parts is sometimes used as a broad measure of morphological complexity^33,34^. The density of connections measures the relative amount of such physical connections in relation to the amount theoretically possible if all parts were connected among them, which is used as a more precise or fine-grained proxy measure of morphological complexity^12,13^. The mean clustering coefficient measures the number of triangular motifs, that is, there nodes connected among them, which captures the level of integration among parts (C = 0, loosely integrated; C = 1, fully integrated). The mean path length measures the distance, in number of links, separating two anatomical parts, regardless of the geometric distance between them, which also co-captures the level of integration of the entire system in terms of effective proximity (L = 1, all parts closely related; L > 1, parts more distantly related. Finally, the heterogeneity of connections measures the difference in the actual number of connections among all anatomical parts, that is, whether all parts connect to the same number of parts (H = 0) or not (H ≠ 0); differences in heterogeneity are linked to differentiation of anatomical parts and, as such, heterogeneity is interpreted as a proxy of anisomerism. Together, these parameters define the overall structure of the system and have been useful in describing anatomical system in various previous works in developmental, functional, and evolutionary contexts. Network parameters were measured in R^35^ using functions from the package igraph^35,36^.

#### Modularity analysis

For the present work, we delimited connectivity modules using a heuristic algorithm based on short random-walks. The algorithm performs short random walks (steps = 3) through the network, which defines a distance matrix that is then analyzed using a clustering tool that search for the partition having maximum value for the optimal function Q (or modularity)^37^. For further details on the method as applied to anatomical networks see Esteve-Altava^12,13^. The expected error of Q can then be calculated using a jackknife approach where each link is treated as an independent observation. For completeness, we also assessed the statistical significance of the individual modules delimited using a Wilcoxon rank-sum test comparing modules’ internal versus external connections. The null hypothesis being that the nodes of a module are equally connected to nodes within and outside the module; the alternative hypothesis being that nodes in a module are more connected among them than to other nodes outside, which follows the general definition of connectivity module as a group of nodes more densely connected among them than to other nodes outside their module. Rejecting the null hypothesis entails that the module delimited by the algorithm departs in a significant way of what would be expected at random. However, it is important to note that in some cases the nodes found by means of connectivity patterns would have a number of nodes too small for such statistical tests to detect a significant difference. Thus, one cannot just relay on the statistical significance to interpret the biological significance or meaning of the modules found by network algorithms.

## Supplementary information

SI
